# Systematic analyses with genomic and metabolomic insights reveal a new species, *Ophiocordyceps indica* sp. nov. from treeline area of Indian Western Himalayan region

**DOI:** 10.3389/fmicb.2023.1188649

**Published:** 2023-07-20

**Authors:** Aakriti Sharma, Ekjot Kaur, Robin Joshi, Pooja Kumari, Abhishek Khatri, Mohit Kumar Swarnkar, Dinesh Kumar, Vishal Acharya, Gireesh Nadda

**Affiliations:** ^1^Entomology Laboratory, Agrotechnology Division, CSIR-Institute of Himalayan Bioresource Technology (IHBT), Palampur, HP, India; ^2^Academy of Scientific and Innovative Research (AcSIR), Ghaziabad, India; ^3^Functional Genomics and Complex System Lab, Biotechnology Division, CSIR-Institute of Himalayan Bioresource Technology (IHBT), Palampur, HP, India; ^4^Biotechnology Division, CSIR-Institute of Himalayan Bioresource Technology (IHBT), Palampur, HP, India; ^5^Chemical Technology Division, CSIR-Institute of Himalayan Bioresource Technology (IHBT), Palampur, HP, India

**Keywords:** genome, marker components, multigene phylogeny, new taxa, novel species, taxonomy

## Abstract

*Ophiocordyceps* is a species-rich genus in the order *Hypocreales* (*Sordariomycetes, Ascomycota*) depicting a fascinating relationship between microbes and insects. In the present study, a new species, *Ophiocordyceps indica* sp. nov., is discovered infecting lepidopteran larvae from tree line locations (2,202–2,653 m AMSL) of the Kullu District, Himachal Pradesh, Indian Western Himalayan region, using combinations of morphological and molecular phylogenetic analyses. A phylogeny for *Ophiocordyceps* based on a combined multigene (nr*SSU*, nr*LSU, tef-1*α, and *RPB1*) dataset is provided, and its taxonomic status within *Ophiocordycipitaceae* is briefly discussed. Its genome size (~59 Mb) revealed 94% genetic similarity with *O. sinensis*; however, it differs from other extant *Ophiocordyceps* species based on morphological characteristics, molecular phylogenetic relationships, and genetic distance. *O. indica* is identified as the second homothallic species in the family *Ophiocordycipitaceae*, after *O. sinensis*. The presence of targeted marker components, *viz*. nucleosides (2,303.25 μg/g), amino acids (6.15%), mannitol (10.13%), and biological activity data, suggests it to be a new potential source of nutraceutical importance. Data generated around this economically important species will expand our understanding regarding the diversity of *Ophiocordyceps*-like taxa from new locations, thus providing new research avenues.

## 1. Introduction

Mushrooms are widely accepted as macrofungi with distinctive, immense, easily notable, and collectable fruiting bodies, serving distinct ecological and economic functions. However, few mushrooms have been reported as toxic (Horowitz, 2015; Govorushko et al., 2019; Tran and Juergens, 2022) due to the presence of harmful secondary metabolites and heavy metals, especially arsenic (Li Y. et al., 2019). Many new species of medicinal mushrooms have been identified, based on morphological characteristics combined with phylogenetic approaches. Ancient oriental customs have emphasized the significance of different medicinal mushrooms with prominent bioactive components which are being valued throughout the world. Among these, the genus *Ophiocordyceps* is one of the most fascinating insect-fungal associations deciphering immense nutraceutical and therapeutic potential.

*Ophiocordyceps* (*Ophiocordycipitaceae, Hypocreales, Ascomycota*) is the largest genus of entomopathogenic fungi having 299 records in Index Fungorum (http://indexfungorum.org/Names/Names.asp; Sung et al., 2007; Xu et al., 2022), and their numbers are enormously increasing. Extensive work by researchers in this area has enhanced the species diversity within the *Ophiocordycipitaceae* family (Araújo et al., 2015, 2018; Shrestha et al., 2019; Chen et al., 2020, 2021; Kuephadungphan et al., 2020, 2022; Mongkolsamrit et al., 2020; Wang et al., 2020; Xiao et al., 2023). Recently, many new species of *Ophiocordyceps* have been identified from different hosts, for example, *O. asiana* (Khao-ngam et al., 2021), *O. bidoupensis* (Zou et al., 2022), *O. buquetii* (Mongkolsamrit et al., 2023), *O. flavida* (Mongkolsamrit et al., 2021), *O. hydrangea* (Zou et al., 2022), *O. laotii* (Mongkolsamrit et al., 2023), *O. mizoramensis* (Chawngthu et al., 2021), *O. puluongensis* (Xu et al., 2022), *O. tessaratomidarum* (Khao-ngam et al., 2021), and *O. vespulae* (Long et al., 2021). These species have been described based on multifarious approaches including micro- and macro-morphological characteristics, multigene phylogeny, and whole-genome analyses combined with metabolomic studies. Simultaneously, over the past years, many endeavors have been made to study the evolutionary and pathogenicity mechanisms within the *Ophiocordyceps* genera (Xia et al., 2017).

To date, among all the studied *Ophiocordyceps* spp., *O*. *sinensis* (Berk.), synonym *Cordyceps sinensis*, commonly known as “Himalayan Viagra” (Yarsagumba, Winter Worm Summer Grass) and Dong Chóng Xià Cǎo in Chinese, is world's most expensive and extremely rare natural medicinal resource. It has a very restricted habitat in the harsh environment of alpine meadows (3,000–5,500 m **a**bove **m**ean **s**ea **l**evel, AMSL) of China, Tibet, Bhutan, Nepal, and India (Li et al., 2011). As a result, harvesting this rare and valuable mushroom is highly challenging and even dangerous as the collectors have to travel through tough terrains. In India, it is known as Keeda Jari or Keeda Ghas and is reported from Uttarakhand (U.K.), Sikkim, and Arunachal Pradesh (Joshi et al., 2018). *Ophiocordyceps sinensis* is commonly recognized as “soft gold” (Woodhouse et al., 2014), “fungal gold” (Panicker, 2017), or “organic gold” (Pouliot et al., 2018), and reported to be even pricier than gold (Li et al., 2015). Owing to the findings of bioactive components in relation to anti-aging, anti-tumor, antioxidant, anti-inflammatory, and immuno-modulatory activities, *Ophiocordyceps* has attracted substantial interest in the modern medicine sector, in addition to its traditional consumption (Lou et al., 2019; Li Y. et al., 2021; Zhang et al., 2021). There was a 900% rise in its price between 1998 and 2008, and top-quality *Ophiocordyceps* were sold at $145,000/kg (Li X. et al., 2019; Li Y. et al., 2021). In addition, its high price and demand led to overharvesting, overexploitation, damage to its fragile mountain environment, and a sharp drop in its yield, which brought it close to extinction (Hopping et al., 2018). Therefore, conservation, sustainable harvesting, and identifying its alternatives are strongly advocated and attempts were made for its *in vivo* cultivation (Zhou et al., 2014; Qin et al., 2018; Li X. et al., 2019).

Nevertheless, *in vitro* cultivation of its anamorph *viz*. *Hirsutella sinensis* and its associated fungus, *Samsoniella hepiali* (syn. *Paecilomyces hepiali*), have been achieved by various researchers. As the research into *Ophiocordyceps* has progressed, new products, such as capsules, powder, and solutions, have been introduced in the global market (Zhou et al., 2009; Au et al., 2012; Hopping et al., 2018; Yang et al., 2018). Some of the products are Pure Cordyceps^TM^ (Aloha Medicinals Inc.), Jin Shui Bao (Jiangxi Jinshuibao Pharmaceutical Co. Ltd.), *Cordyceps* capsule (Geneferm Biotechnology Co. Ltd.), and Bailing capsules (Hangzhou East China Pharmaceutical Co., Ltd.; Zhou et al., 2009; Elkhateeb and Daba, 2022). In India, wild *O. sinensis* and some mycelial-based products are being used as nutraceuticals and immunity boosters (Sharma, 2004; Panda and Swain, 2011; Maity, 2013). However, the demand, market, and price for these products are cheaper than the wild intact *Ophiocordyceps*.

Keeping in view the challenges and potential of *O. sinensis*, the identification, and positioning of any *Ophiocordyceps* species from the wild inhabiting low-lying and approachable areas is the need of the hour. Under these circumstances, the present study was planned, and exploratory surveys were conducted between 2015 and 2021 in different districts (Kangra, Chamba, Mandi, Kullu, and Lahaul and Spiti; 1,000–4,800 m AMSL) of Himachal Pradesh (H.P.) India, to explore the presence of any *Ophiocordycep*s species of commercial importance. During our surveys, one kind of unknown pathogenic fungus infecting larvae of *Thitarodes* sp. buried in soil was collected from tree line locations of Kullu, H.P., Indian Western Himalayas. The present study was conducted with the objective to confirm the taxonomic position of the collected specimens, and we introduced a new taxon of *Ophiocordyceps* of commercial importance from low-height areas (2,202–2,653 m AMSL) of Himachal Pradesh, India, with morphological descriptions coupled with multigene phylogeny, genomic, and metabolomic estimations.

## 2. Materials and methods

### 2.1. Sampling

**Figure 1:**
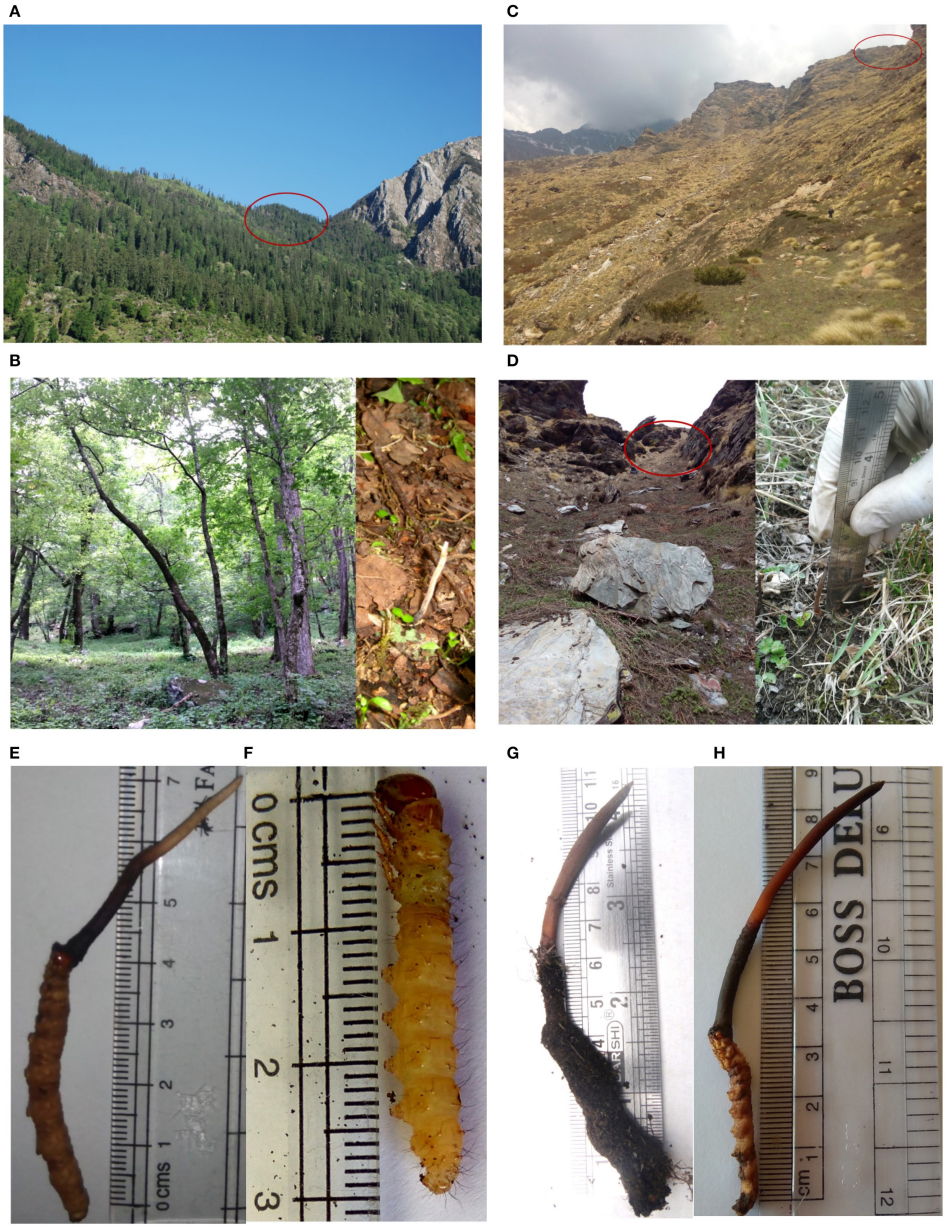
Comparative account of habitat and morphology of *Ophiocordyceps indica* (HP) and *O. sinensis* (UK), India. **(A, B)** Habitat of *O. indica* in HP, India (2,455 m AMSL). **(C, D)** Habitat of *O. sinensis* in UK, India (4,015 m AMSL). **(E)** Immature *O. indica*. **(F)** Host larva (live) of *O. indica*. **(G, H)** Immature *O. sinensis*.

**Figure 2:**
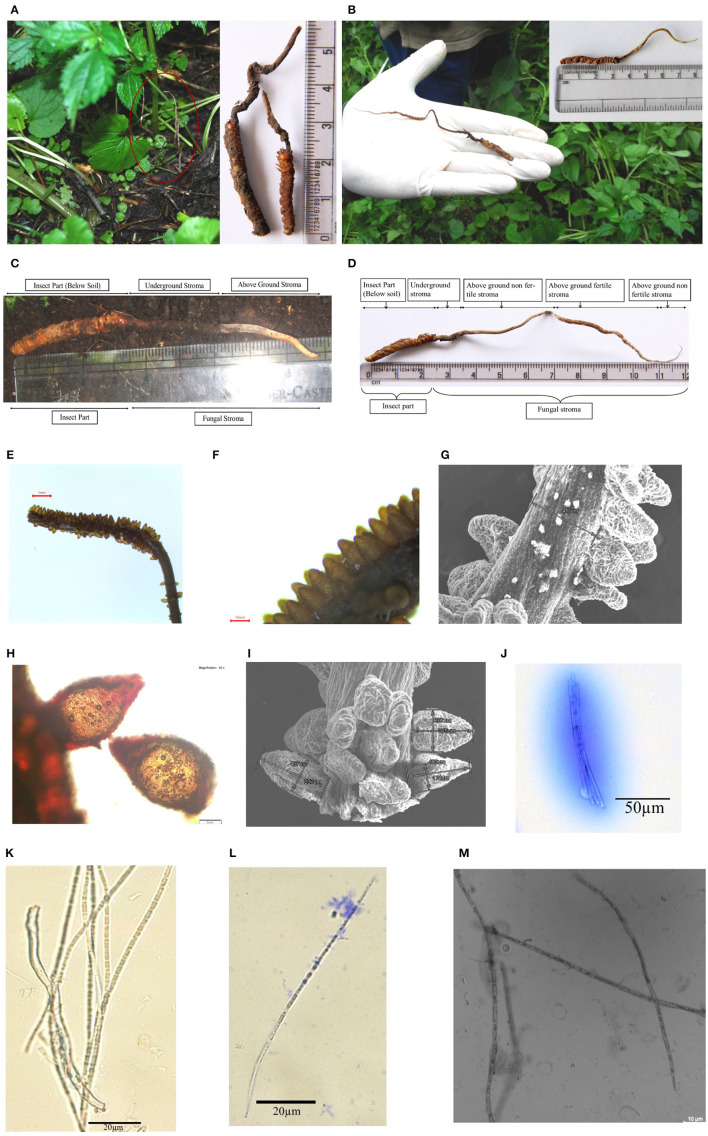
Habitat and morphology of *Ophiocordyceps indica*. **(A)** Matured stroma visible above the ground under the trees, surrounded with dense vegetation. **(B)** Stroma emerging from head between two eyes of lepidopteran larva. **(C)** Freshly collected immature *O. indica*. **(D)** Mature *O. indica*. **(E, F)** Fertile part of the stroma. **(G)** SEM micrograph of fertile part of *O. indica* showing stalk size and arrangement of perithecia. **(H)** Perithecium. **(I)** SEM of perithecia showing size and arrangement. **(J)** Ascus with ascospores. **(K–M)** Filiform ascospores with part spore. *Bars* E, 1 mm; F, 10 mm; G, 500 μm; H, 20 μm; I, 500 μm; J, 50 μm; K–L, 20 μm; M, 10 μm.

**Figure 3:**
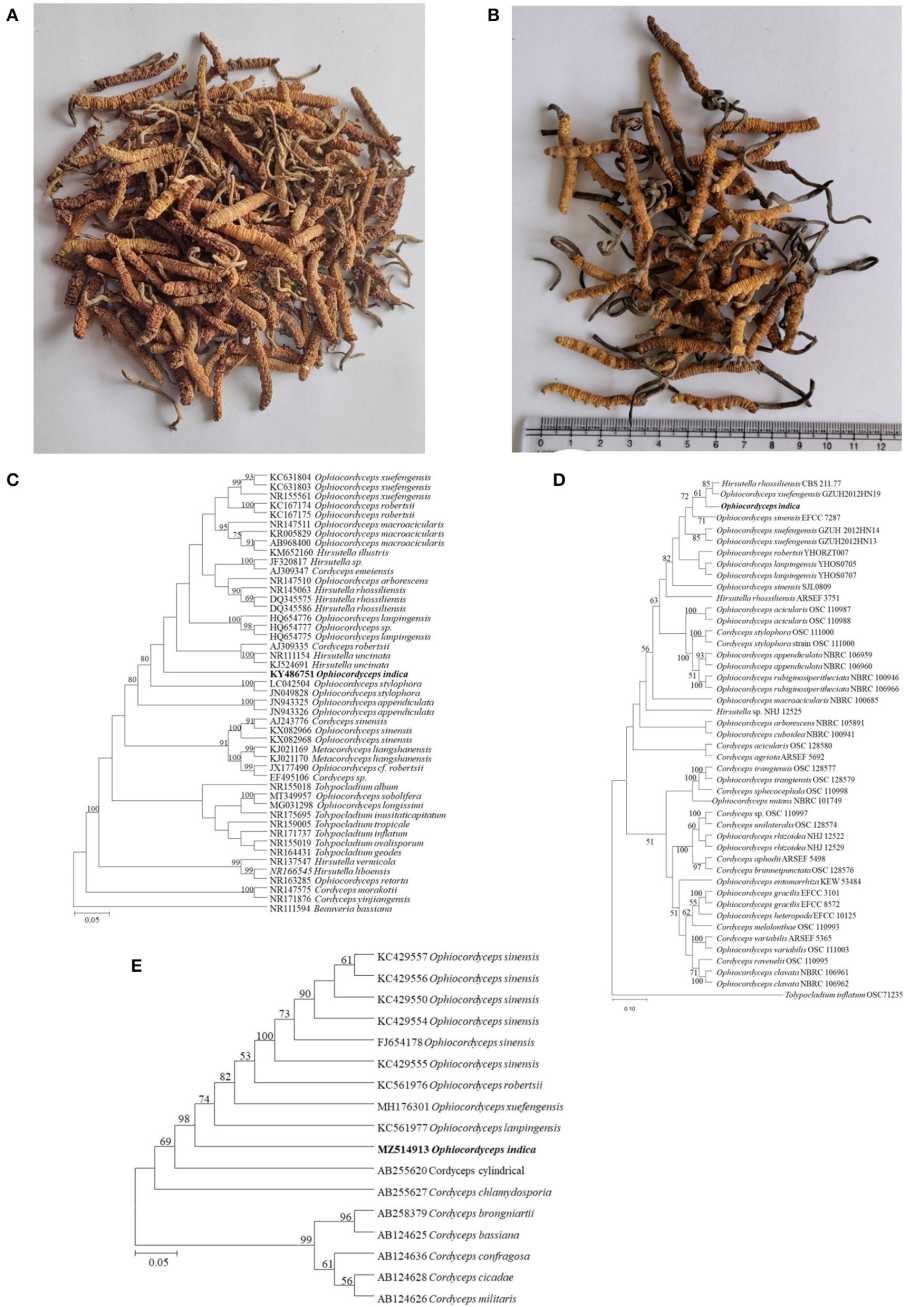
*Ophiocordyceps* samples used in the present study and phylogenetic studies. **(A)** *O. indica* from HP. **(B)** *O. sinensis* from local Indian market. **(C)** Neighbor-joining (NJ) tree of *Ophiocordyceps indica*, determined on the basis of ITS region with a bootstrap value of 1,000. *B. bassiana* was selected as outgroup taxa. **(D)** Phylogenetic tree of *O. indica* with other species of *Ophiocordyceps* with maximum likelihood (ML) method based on the combined data set of *SSU, LSU, RPB1*, and *TEF-1*α with log-likelihood -40,948.921. *T. inflatum* was selected as outgroup taxa. Numbers above the branches are bootstrap values based on 1,000 replicates, ML bootstrap support values >50%. **(E)** Neighbor-joining (NJ) tree based on *MAT 1-2-1* gene region with a bootstrap value of 1,000.

Samples of *O. indica* were observed and collected from the tree line areas near Village Manihar (2,455 m AMSL; 31°53′38.3”N 77°19′10.2”E; 2,479 m AMSL; 31°54′21.4360”N 77°19′02.0569”E) and near Panchanala (2,299 m AMSL; 31°53′38.5869”N 77°20′49.3790”E) of Kullu District, H.P., India, on 13^th^ and 14^th^ July 2016, respectively (Figures 1A, B, 2A–D; Supplementary Video 1; fgcsl.ihbt.res.in/Ophiocordyceps_indica). Samples were observed and collected for the first time at 2,479 m AMSL (Figures 2A–D) in July 2016. In subsequent years, specimens were further recorded and studied in almost similar habitats at different locations and altitudes (2,202–2,653 m AMSL) in the same district (Figure 3A; Supplementary Videos 2, 3). Interestingly, the morphology and habits of these specimens were somewhat similar to *O. sinensis* (Joshi et al., 2018), despite differences in their habitats, i.e., treeline (Figures 1A, B, E) and alpine meadow (Figures 1C, D, G) distributions, respectively. Its occurrence at low-height treeline areas, in contrast to the alpine meadow distribution of *O. sinensis*, triggered us to study and bioprospecting this important fungal species. During surveys, only the fruiting body or stroma of varying sizes (up to 12 cm) of *O. indica* was visible above blackish soil (pH 5.50–6.07) covered with decayed leaves (Figures 2A–C). While digging the soil, stroma was found protruding from the head of a vertically positioned dead lepidopteran caterpillar (Figures 1E, 2A–D). Samples were collected, and soil was removed gently using a soft brush (Supplementary Video 4). *Ophiocordyceps* which were marketed as *O. sinensis* in India were studied in its natural habitat and collected from the Rilkot area (4,015 m AMSL; 30°18′40.7599”N 80°11′31.5069”E) of district Pithoragarh (Uttarakhand), India (Figures 1C, D), on 17^th^ May 2016, and some were procured locally. The samples were stored at -80 and 4 °C, and some samples were dried at room temperature after careful cleaning with a brush. Some specimens were stored at CSIR-Institute of Himalayan Bioresource Technology (IHBT), Palampur, and the holotype is deposited at Central National Herbarium, Howrah, Kolkata, India, GN-22311 (CAL 1880). *Ophiocordyceps* samples collected from Uttarakhand were used as reference material for habit and habitat comparative studies and referred to as *O. sinensis* in the manuscript (Figure 3B).

### 2.2. Morphological studies

The specimens were collected along with their host, the larva of Lepidoptera, and were kept in plastic bags and air-tight containers. Macromorphological characteristics were recorded during the collection time. The specimens were preserved by air drying followed by deep freezing and were stored in the Entomology Laboratory, CSIR-IHBT with voucher numbers. Furthermore, the specimens were studied and identified based on macroscopic and microscopic characteristics using stereo microscope (Magnus MSZ-Bi, Noida, India), compound microscope (BX53, Olympus, Tokyo, Japan), confocal microscope (Leica STELLARIS 5, Mannheim, Germany), and scanning electron microscope (S-3400 N, Hitachi, Tokyo, Japan). Furthermore, water-mounted and lactophenol cotton blue-stained slides of perithecia, asci, and ascospores were observed and photographed.

### 2.3. Scanning electron microscopy (SEM)

Surface morphological studies of *O. indica* were conducted using SEM. In brief, a small part of the fruiting body was cut with a fine sterile blade, washed with distilled water, and placed on the double-sided carbon tape mounted on the aluminum stub. The mounted sample was left at room temperature, in dust-free condition for drying. Then, this dried sample was coated with gold using a sputter coating unit (E1010 Ion Sputter Hitachi, Tokyo, Japan) at 10 Pa vacuum for 10 s. The images were captured in SEM mode at desired magnifications.

### 2.4. Library preparation, sequencing, and phylogenetic analyses

High-quality genomic DNA was isolated from ~40 mg stroma of *O. indica* specimen using Wizard Genomic DNA Purification Kit (Promega, Madison, USA) and checked on 0.8% agarose gel. ITS, nr*SSU*, nr*LSU, RPB1*, 3′ end of *TEF-1*α, and *MAT 1-2-1* were, respectively, amplified with primer pairs described by Chen et al. (2013) and sequenced on a 3730xl DNA Analyzer (Applied Biosystems, CA, USA) *via* ABI Big-Dye version 3.1 sequencing kit (Applied Biosystems, CA, USA) as per the manufacturer's instructions. The resulting chromatograms were evaluated using Chromas Lite, and a homology search was done using NCBI-BLAST (http://blast.ncbi.nlm.nih.gov). The sequences used for the alignment were downloaded from NCBI and were aligned with Clustal W (Thompson et al., 2003). Pairwise distance matrices and phylogenetic consensus trees were analyzed using MEGA11 software (Molecular Evolution Genetic Analysis; Tamura et al., 2021). The taxon information and GenBank accession numbers used in the molecular analyses are listed in Supplementary Table 1.

Forty-five ITS sequences (Supplementary Table 1A) of *Ophiocordyceps* species, closely related to *O. indica*, were used to align and construct a neighbor-joining (NJ) tree (Tamura et al., 2021). NJ estimation of the phylogeny of ITS was performed with 1,000 bootstrap replicates by MEGA11. *Beauveria bassiana* was selected as outgroup taxa. The Tamura-3-parameter+Gamma distribution with invariant sites nucleotide substitution model was used for the substitution model (Chen et al., 2013). NJ was also constructed for *MAT 1-2-1* gene using Kimura 2 parameter nucleotide substitution model. Furthermore, for the construction of a multigene tree, each gene region was independently aligned and improved manually and nr*SSU*, nr*LSU, RPB1*, and 3′ end of *TEF-1*α gene sequences were combined to form a dataset. The four gene datasets from the *Ophiocordyceps* species and datasets obtained from GenBank were then aligned using MUSCLE in MEGA11 (Tamura et al., 2021). Alignments were manually adjusted to allow maximum sequence similarity. Gaps were treated as missing data. Unweighted maximum parsimony (MP) and maximum likelihood (ML) analyses were performed using PAUP^*^ 4.0a10 (Swofford, 2002). The heuristic search option with TBR branch switching and 1,000 random sequence additions was used to infer trees. Maxtrees were 5,000 branches of zero length collapsed, and all multiple parsimonious trees were saved. Clade stability of the trees resulting from the parsimony analyses was assessed by bootstrap analysis with 1,000 replicates, each with 10 replicates of random stepwise addition of taxa (Felsenstein, 1985). Trees were saved and exported to graphics programs. *Tolypocladium inflatum* was selected as outgroup taxa. Clades that were supported with 50% or greater values were considered significant, while the values below 50% were not displayed in the phylogenetic tree. Furthermore, DNA libraries were constructed with PacBio SMRTbell template preparation kit. SMRTbell template library was checked with Bioanalyzer DNA 12,000 chip (Agilent Technologies, Santa Clara, CA, USA). PacBio Single Molecule Real-Time DNA sequencing technology developed by Pacific Biosciences was considered with 51X coverage for the sequencing of the *O. indica* genome. The assembly of the long reads obtained was tested on various assemblers and compared to obtain the best assembly of the genome.

### 2.5. Isolation of RNA, sequencing, and assembly for transcriptome studies

Fresh fruiting bodies of *O. indica* collected from two different locations were used for the transcriptome study. After collection, these samples were immediately frozen in liquid nitrogen and stored in the laboratory at -80 until further processing. One hundred milligrams of *O. indica* sample was ground into a fine powder in liquid nitrogen using a mortar and pestle and processed using IRIS method (Ghawana et al., 2011). The purity and concentration of RNA were assessed by determining the absorbance of the sample at 260 and 280 nm using a spectrophotometer (Thermo Scientific, Wilmington, DE, USA) and Agilent Bioanalyzer Chip 7500 Series II (Agilent Technologies, Santa Clara, CA, USA). The RNA was resolved on the gel. The RNA sequencing library was prepared with mRNA-seq 8 sample preparation kit (Illumina, San Diego, CA, USA) using random primers. The concentration ≥800 ng/μl of RNA was used for the preparation of the cDNA library and was run on NovaSeq 6000 Sequencing System (Illumina, San Diego, CA, USA).

### 2.6. Assembly and functional annotation

The *O. indica* fungal genome was first assembled from the most established long-read technology, Pacific Biosciences (PacBio, CA, USA), by using different assemblers: Celera (Denisov et al., 2008; PbcR) and Canu. The best assembler, Canu pipeline along with SSPACE (Boetzer et al., 2011) was used to do an independent whole-genome assembly of raw reads using default parameters to generate the scaffolds. Finally, the completeness of *O. indica* genome assembly was assessed by the presence of BUSCO orthologous genes [Benchmarking Universal Single-Copy Orthologs; (http://gitlab.com/ezlab/busco)], with the default values. BUSCO uses a set of universal single-copy orthologs. The assembled transcriptome was then aligned with assembled genome using GMap. The protein-coding genes in *O. indica* were generated by means of the fully automated Fungal Genome Annotation Pipeline (FunGap; Finn et al., 2016). FunGap predicted gene models in three major steps: (a) pre-processing, (b) gene prediction, and (c) evaluation and filtration. To attain high-quality gene models, the program FunGAP: Fungal Genome Annotation Pipeline (https://github.com/CompSynBioLab-KoreaUniv/FunGAP) was used, where it takes two inputs: *O. indica* genome assembly in FASTA format and mRNA sequencing reads of *O. indica* in FASTQ format. This program incorporates multiple gene predictors, *viz*., Augustus, Braker, and Maker (Stanke et al., 2006; Brandi et al., 2008; Hoff et al., 2016) that assessed all the computationally predicted genes and assemble the best gene model for those particular genes which have shown highest homology to known sequences.

The best score for all predicted gene models of *O. indica* fungus from FunGAP was then selected on the basis of the alignment of translated protein sequences with well-known functional tools, *viz*., Pfam, BUSCO, and BLAST (Boratyn et al., 2013). The alignment of the transcriptome, Ascomycota BUSCOs, and homologous peptide to our gene predictions was carried out to evaluate the quality assessment of predicted gene models. InterProScan version 5.16.55 (Zdobnov and Apweiler, 2001) was then used to analyze the functional annotation of the predicted gene models. Non-coding RNA (ncRNA), including transfer RNA (tRNA), ribosomal RNA (rRNA), small nuclear RNA (snRNA), and small nucleolar RNAs (snoRNA), were predicted using cmsearch in Infernal using the Rfam library Rfam.cm.1_1 with the hits selected above the e-value threshold of 1e-5. To identify the six types of SSRs within the *O. indica* genome, we used the MISA Perl script (http://pgrc.ipk-gatersleben.de/misa/). The minimum repeat unit size for mono-nucleotides was set at 10 and six for di- and at five for tri- to hexa-nucleotides. RepeatModeler-open-1.0.11 (http://www.repeatmasker.org/RepeatModeler) to detect transposable elements in *O. indica* was used. Furthermore, for functional analysis of predicted gene models, we assigned interproscan-5.28-67.0 (https://github.com/ebi-pf-team/interproscan/wiki) to protein-coding genes of the *O. indica* genome. It generated resultant files in two different formats (.gff and.tsv) containing annotated models. Furthermore, Pfam annotations were used to identify unique or enriched functional domains in the *O. indica* genome. MCScanX package was used to generate the collinear blocks between *O. sinensis* and *C. militaris*. In brief, a total of 7,939 protein sequences of *O. sinensis* together with its genomic annotation gff3 file were downloaded from JGI Genome Portal. BLASTP was then used to align them to the gene models of *O. indica* with an e-value of 1e-10. To make MCScanX produce reasonable results, the number of hits from BLASTP for each gene was set to 5 as suggested by the MCScanX manual. Based on the gene positions shown in the gff3 files, MCScanX was finally used to detect the collinear blocks between *O. sinensis* and *O. indica*. To recognize the peroxidase genes, the protein sequence profiles for each peroxidase class for 12 entomopathogenic fungi, comprising *O. australis, O. camponoti-rufipedis, O. indica, O. polyrhachis-furcata, O. sinensis, O. unilateralis, B. bassiana, C. militaris, H. sinensis, M. acridum, M. anisopliae*, and *T. inflatum*, were fetched from the Fungal Peroxidase Database (fPoxDB; http://peroxidase.riceblast.snu.ac.kr). To identify the proteases in *O. indica*, all protein sequences of peptidase from the MEROPS database were downloaded (Release 10.0; http://merops.sanger.ac.uk/index.shtml), which uses a hierarchical and structure-based method to classify the peptidases. The blastp searches were implemented against MEROPS peptidase with a cutoff E-value of 1e-20 to detect the proteases in *O. indica*. We detected transporters in *O. indica* by means of blastp searches against the Transport Classification Database with a cutoff E-value of 1e-03 (http://www.tcdb.org/). Blastp searches against the KinBase database (http://kinase.com/web/current/kinbase/) with a cutoff E-value of 1e-10 to identify Kinases were also performed. To investigate CAZymes, we searched our fungus against dbCAN HMMs using HMMER3. For this, “dbCAN-fam-HMMs.txt” was downloaded from the dbCAN database (https://bcb.unl.edu/dbCAN2/index.php), followed by HMM searches using “hmmscan.” Then, the search results were finally parsed by the script of “hmmscan-parser.sh” implemented in the dbCAN database. The Orthology assignment of the *O. indica* genome was carried out by means of the OrthoFinder-2.2.1 (https://github.com/davidemms/OrthoFinder) package. The final annotation set for the *O. indica* genome was compared with other fungal genomes to characterize orthologous relationships. First, the following annotation sets were extracted from genomic databases for the 18 fungal species: (i) insect–fungal species: *O. australis, O. camponoti-rufipedis, O. polyrhachis-furcata, O. sinensis, O. unilateralis, B. bassiana, Cordyceps militaris, H. sinensis, Metarhizium acridum, M. anisopliae*, and *T. inflatum*, (ii) plant–fungal species: *Botrytis cinerea, Fusarium graminearum, Grosmannia clavigera, Magnaporthe grisea, Sclerotinia sclerotiorum*, and *Verticillium alfalfa*, and (iii) outgroup: *Saccharomyces cerevisiae*. Orthology assignment was carried out with the Orthofinder pipeline (Emms and Kelly, 2015; https://github.com/davidemms/OrthoFinder) to reveal rooted gene trees for all ortho groups and infers a rooted species tree for the species being analyzed. Phylogenetic analysis using the PROTGAMMA amino acid substitution model in RAxML (Stamatakis, 2014) was performed with 1,000 bootstrap replicates. To determine gene families in the analyzed *O. indica* genome that had experienced remarkable expansion or contraction, CAFE-4.0.2 (The Computational Analysis of Gene Family Evolution; https://hahnlab.github.io/CAFE/) was executed. CAFE was run with default parameters accessing the lambda parameter (option -s) with a *P-*value cutoff of 0.05 (option -p). Viterbi *P*-values were enumerated for each significant ancestry to evaluate significant expansion or contraction across a definite branch.

### 2.7. Metabolite profiling

Different extraction methods *viz*., cold water, hot water, and methanol (50:50, 80:20, and 100%) were tried for the extraction of *O. indica* and *O. sinensis* samples using an ultrasonic water bath (Elma S 300 H, Elma Schmidbauer GmbH, Singen, Germany). Metabolomic studies were done using UHPLC-Q-ToF-IMS (Agilent, Santa Clara, USA) with an Eclipse Plus C18 RRHD column (Joshi et al., 2018). The identification of unknown metabolites other than targeted ones was searched using acquired data against the METLIN database. Identified metabolites were classified according to their biochemical groups namely, sugars, fatty acids, amino acids and their derivatives, nucleosides, and others. Amino acids (Agrawal et al., 2019) and mannitol were quantified in hot water extract using UPLC.

### 2.8. Antioxidant activities

DPPH and ABTS radical-scavenging activities of hot water extracts of *O. sinensis* and *O. indica* were performed (Blois, 1958; Joshi et al., 2015, 2018). The reaction was performed using different concentrations of each sample (1–10 μl) by adding 2.9 mL of 0.06 mM DPPH solutions to the reaction mixture and vortexed. All the tubes were covered with aluminum foil to carry out the reaction in the dark. The reaction mixture was incubated for half an hour at 25 °C, and the absorbance was recorded at 517 nm wavelength. Similarly, the ABTS assay was performed using an aqueous solution of 2,2′-azinobis-(3-ethyl-benzothiazoline-6-sulphonate; ABTS, 7 mM, 5 mL) and potassium persulphate solution (88 μL, 140 mM) which were mixed and kept overnight in the dark to generate ABTS^.+^ cation (2.45 mM). ABTS^.+^ solution is diluted with ethanol to achieve an absorbance of 0.7 ± 0.05 at 734 nm. The reaction was performed using different concentrations of each sample (1–10 μl) which were mixed with 2 mL of diluted ABTS^.+^ radical solution, and the absorbance was recorded at 734 nm after 6 m of initial mixing. The experiments were carried out in triplicates, and IC_50_ values were calculated by plotting a graph between absorbance and concentration.

### 2.9. Cell viability studies

The cellular viability of peritoneal macrophages in response to hot and cold water extracts of *O. indica* was assessed using four different concentrations (25, 50, 100, and 200 μg/mL; Sharma et al., 2018).

The viability of cells was determined using the following equation:


$$\begin{array}{l} \text{Cell viability }\left(\text{\%}\right)=\left(\text{Test sample}\right)/\left(\text{Control sample}\right)\times \;100 \end{array}$$

where test sample = OD of cells treated with tested samples and control sample = OD of cells treated with only media.

#### 2.9.1. Nitrite production

The peritoneal macrophages (0.5 × 10^6^) were supplemented with four non-lethal concentrations (25, 50, 100, and 200 μg/mL) of hot and cold water extracts *O. indica* and stimulated with LPS (1.5 μg/mL; Sigma Aldrich, St. Louis, USA) for 16 h in a CO_2_ incubator. The supernatant was collected after incubation, and NO production was analyzed using Griess reagent as per the manufacturer's protocol (Promega, USA; Sharma et al., 2018).

### 2.10. Statistical analyses

The data were statistically analyzed using the SPSS software (SPSS version 27.0.1). Student's *t*-test was used to determine and compare the means (McDonald, 2014). Differences were considered statistically significant at a *P*-value of ≤ 0.05 and a *P*-value of ≤ 0.001.

## 3. Results

### 3.1. Taxonomy

*Ophiocordyceps indica* G. Nadda & A. Sharma, sp. nov. Figure 2.

**Index Fungorum Number:** IF559266.

**Etymology:** Named after the country location (India), from where this species was collected.

**Host:** *Thitarodes* sp.

**Habitat:** On the larva of Lepidoptera (*Thitarodes* sp.) buried in the soil.

**Distribution:** Kullu, Himachal Pradesh, India.

**Holotype:** Collected from village Manihar (2,479 m AMSL; 31°54′21.4360”N 77°19′02.0569”E) of Kullu District, Himachal Pradesh, INDIA, on the lepidopteran larva, 13 Jul. 2016. G. Nadda, GN-22311 (CAL 1880, Holotype). GenBank: ITS KX679571; *SSU* MZ571406; *LSU* KY486751; *TEF*-*1*α MZ514914; *RPB1* MZ514912; *MAT 1-2-1* MZ514913.

**Sexual morph:** Stroma solitary, simple, or branched (up to 120 mm long above soil, dia 0.42–1.70 mm and 6–24 mm below soil), protruding from head between two eyes of host caterpillar (20–36 mm; Figures 2A–D). Matured stroma was thin, slender and cylindrical, fibrous, curved more or less, and light brown in color (Figures 2D, E; Supplementary Videos 1, 3). Perithecia brown, crowded, oval- to flask-shaped, superficial 312–467 × 174–350 μm (*n* = 30), loosely packed and arranged at right angles to the surface of stroma, followed by tapering part (Figures 2F–I). Asci cylindrical, filiform, thread-like 150.0–382.2 × 7.58–12.1 μm (*n* = 20; Figure 2J); Ascospores filiform, elongate thread-like 68.0–307.7 × 3.03–5.54 μm (*n* = 20), multiseptate, hyaline, not easy to break into part-spores; part-spores cylindrical (Figures 2K–M).

**Asexual morph:** Undetermined.

**Comments:** *Ophiocordyceps indica* specimens were observed and collected from tree line locations of Kullu, H.P., India (Figures 1A, B, E, 2A–D; Supplementary Video 1). Morphologically, the caterpillar part and immature stroma of *O. indica* showed some similarity with *O. sinensis;* however, the caterpillar was dark brownish in the former (Figure 1E; Supplementary Video 2) than yellowish golden in the latter (Figure 1H). We observed and collected a live caterpillar from the field (Figure 1F; Supplementary Video 5) but were unable to rear it under laboratory conditions.

**Specimens examined:** Immature and mature specimens (lepidopteran larva and fruiting body) collected from Manihar (2,479 m AMSL; 31°54′21.4360”N 77°19′02.0569”E) of Kullu District, Himachal Pradesh, INDIA, 13^th^ July 2016, GN-23701 (CAL), GN-23702 (CAL), and Panchanala (2,299 m AMSL; 31°53′38.5869”N 77°20′49.3790”E) of Kullu District, Himachal Pradesh, INDIA, 14^th^ July 2016, GN-23703 (CAL) GN-23704 (CAL).

### 3.2. Genome sequencing, assembly, and evaluation

ITS has been used as an important molecular marker in the identification and genetic analysis of fungi including *Ophiocordyceps* species. In the present study, molecular data and systematic analyses based on the ITS region of the *Ophiocordyceps* specimen (KX679571) revealed its 97.67% similarity with *Hirsutella uncinata* (Figure 3C). Furthermore, a multigene phylogeny study based on nr*SSU* (MZ571406), nr*LSU* (KY486751), *RPB1* (MZ514912), 3′ end *TEF*-*1*α (MZ514914), and *MAT 1-2-1* (MZ514913) revealed it to be a novel species of the genus *Ophiocordyceps* (Figures 3D, E). The four-gene combined dataset consisting of 2,828 base pairs (nr*SSU* 812 bp, nr*LSU* 635 bp, *TEF*-*1*α 911 bp, and *RPB1* 470 bp) of *O. indica* were used to build a multigene phylogenetic tree of closely related species of *Ophiocordyceps* (Figure 3D; Supplementary Table 1B). It is evident that *O. acicularis, O. indica, O. lanpingensis, H. rhossiliensis, O. sinensis*, and *O. xuefengensis* were independent species in the four gene tree. The combined dataset (nr*SSU*, nr*SU, TEF*-*1*α, and *RPB1*) comprised 8,191 characters after alignment, of which 5,496 were constant, 1,483 as parsimony uninformative, and 1,212 as parsimony informative. Parsimony analyses generated 1,000 trees; one of most appropriate has tree length = 5,881, CI = 0.5810, RI = 0.6279, RC = 0.3648, and HI = 0.4190. ML tree of the same dataset resulted in a tree with a log-likelihood of -40,948.921 is presented (Figure 3D). The matrix had 2,212 distinct patterns, and the parameters GTR model were as A = 0.237939, C = 0.271626, G = 0.281296, and T = 0.209139. Furthermore, the sequence of the *MAT 1-2-1* gene of *O. indica* was 337 bp which was aligned with 16 sequences of *Ophiocordyceps* species to construct a neighbor-joining tree (Figure 3E; Supplementary Table 1C). Based on the *MAT 1-2-1* gene tree, *O. lanpingensis, O. robertsii, and O. sinensis* are observed to get clustered closely to *O. indica*. The new species, *O. indica* was clearly phylogenetically distinct from the other known species. Additionally, whole-genome sequencing was done that revealed 94% genetic similarity with *O. sinensis*, confirming it to be a novel species of the genus *Ophiocordyceps*. Based on phylogenetic analyses of the whole genome, *O. indica* is found closely associated with *O. sinensis* followed by *H. sinensis*.

**Figure 4:**
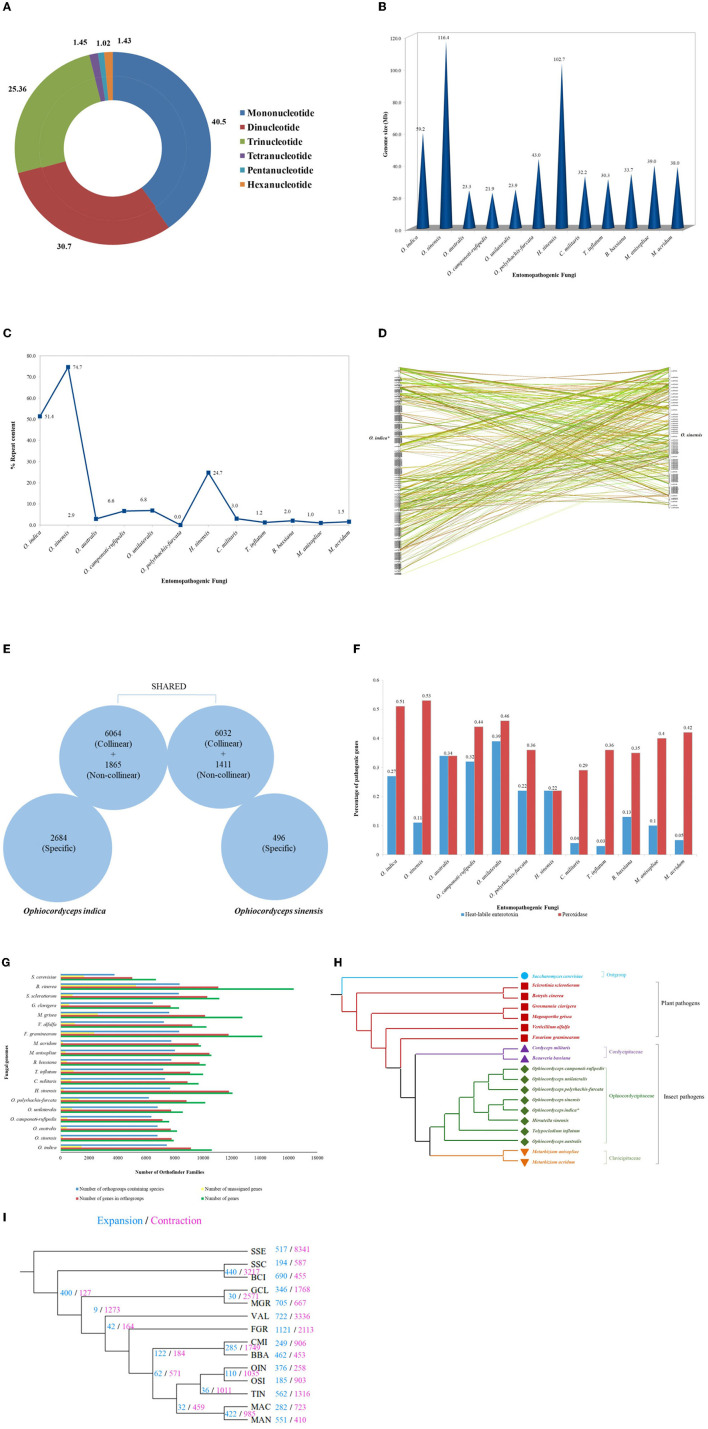
Genetic studies of *O. indica*. **(A)** Overview of SSRs identified in *O. indica* genome displaying proportion of six types of SSRs from mono-nucleotides to hexa-nucleotides detected by means of MISA Perl script (http://pgrc.ipk-gatersleben.de/misa/). **(B)** Genome size of *O. indica, O. sinensis*, and insect-pathogenic fungal genomes. **(C)** Repeat contents (%) and estimated genome size of *O. indica* and other 11 sequenced insect-pathogenic fungal genomes. **(D)** Synteny between *O. indica* and *O. sinensis* genomes. **(E)** Comparison of genetic similarity. **(F)** Pathogenic proteins of *O. indica* with its closely related fungal genomes. **(G)** Family size distribution among *O. indica* and other fungal genomes. **(H)** Phylogenetic analyses. **(I)** Evolution of expanded and contracted gene families, where numbers of gene families showing expansion (blue) or contraction (pink) for each lineage are indicated on each branch of the phylogenetic tree.

The respective sequences of the whole genome are deposited at the NCBI portal under the BioProject number PRJNA699552 (*O. indica*). The data generated on the PacBio were long reads with an average length of 5,807 bp with ~6.9 Gb in overall 1,182,152 in fasta read sequences with minimum and maximum read lengths of 35 and 51,733 bp, respectively (Accession No. PRJNA699552). Canu assembler (Koren et al., 2017) was found to be the best in terms of the lowest number and size of contigs (356 and 59,047,143 bp) with an N50 contig length of 6,27,704 bp. Finally, 229 scaffolds (13,118 SSRs) were generated after scaffolding with a total size estimated to be 59,247,067 bp and a ScafN50 value of 852,095 bp (Table 1). BUSCO (Simão et al., 2015) [benchmarking universal single-copy ortholog] evaluation of the *O. indica* genome revealed that 95.8 and 3.3% of the 1,315 expected Ascomycota BUSCO conserved genes were identified as complete and fragmented, respectively. A few numbers of missing genes (0.9%) indicated the completeness of the *O. indica* genome (Table 2). Out of these 13,118 SSRs, the most abundant type was mono-nucleotides repeats (5,255; 40.05%), followed by di-nucleotides (4,022; 30.7%), tri-nucleotide (3,328; 25.36%), tetra-nucleotides (191; 1.45%), hexa-nucleotides (188; 1.43%), and penta-nucleotides (134; 1.02%' Table 3 and Figure 4A).

**Table 1:** Comparison of PacBio assembly statistics for *Ophiocordyceps indica* genome using different assemblers.

	**Canu**	**Celera**
**Scaffolds**		
Number of scaffolds	229	607
Total size of scaffolds	59,247,067	54,362,712
Longest scaffold	2,887,237	1,113,332
Shortest scaffold	1,722	3,916
Mean scaffold size	258,721	89,560
Median scaffold size	26,084	40,178
N50 scaffold length	852,095	214,133
**Contigs**		
Number of contigs	356	1,283
Total size of contigs	59,047,143	53,109,470
Longest contig	1,618,075	1,048,886
Shortest contig	1,722	3,246
Mean contig size	165,863	41,395
Median contig size	25,886	15,772
N50 contig length	627,704	144,810

**Table 2:** Completeness of *Ophiocordyceps indica* assembly (PacBio) evaluated by means of the presence of BUSCO orthologous genes.

**Genotype**	**BUSCO fungal lineage**	**Single**	**Duplicated**	**Fragmented**	**Missing**	**Complete (%)**
Genome assembly	Fungi odb9 (290)	286	1	1	2	98.9
	Ascomycota odb9 (1,315)	1,260	4	44	11	95.8
Annotated protein model	Fungi odb9 (290)	286	1	4	0	98.6
	Ascomycota odb9 (1,315)	1,264	51	49	2	96.1

**Table 3:** Occurrence of simple sequence repeats (SSRs) in *Ophiocordyceps indica* fungal genome.

**Repeat type**	**Number**	**Proportion (%)**
Mononucleotide	5,255	40.05
Dinucleotide	4,022	30.7
Trinucleotide	3,328	25.36
Tetranucleotide	191	1.45
Pentanucleotide	134	1.02
Hexanucleotide	188	1.43
**Total**	**13,118**	**100**

To annotate gene models, an average of ~22 Gb of clean RNA-seq (sequencing) data were generated from the Illumina Nova-Seq sequencing platform and obtained 25,558 transcripts (average length 2,096 bp). The assembled transcriptome showed more than 99.46% alignment with a criterion of using >90% in terms of coverage and identity, again indicating a very good quality of the assembled genome. The annotation software, FunGAP (Min et al., 2017), used multiple gene predictor models (Stanke et al., 2006; Brandi et al., 2008; Hoff et al., 2016) along with transcripts as a query finally resulted in 10,613 protein-coding genes. Using BUSCO from Ascomycota lineage, the best-annotated protein models were observed with (1,264 out of 1,315) 96.10% as complete, (51) 3.90% as duplicated, (49) 3.72% as fragmented, and (2) 0.15% as missing, revealing a very good quality of our gene predicted models (Table 2). The gene prediction in *O. indica* produced by FunGap revealed a higher quality and hence can be processed for downstream protein analysis. InterProScan (Quevillon et al., 2005) findings revealed that 69.2% of annotated models had matches in the InterPro database, 6.7% in KEGG, and 49.1% in Gene Ontology (GO; Table 4). Non-coding RNA (ncRNA) genes using Infernal (Nawrocki and Eddy, 2013) yielded 77 ribosomal RNAs, 92 transfer RNAs, 5 small nuclear RNAs, and 1 snoR.

**Table 4:** Annotation of protein-coding genes in *Ophiocordyceps indica* fungal genome.

**Annotated models**	**Protein-coding genes**
GO annotation	5,209 (49.1%)
KEGG pathway	717 (6.7%)
InterPro domain	7,344 (69.2%)

### 3.3. Size comparison and collinearity of *O. indica* with two highly prized entomopathogenic fungal genomes (*O. sinensis* and *Cordyceps militaris*)

The genome of *O. indica* was found to be 59.2 Mb, ~2 times greater than other entomopathogenic fungi (range 21.91–43.0 Mb), *viz*., *O. polyrhachis-furcata* (43 Mb; Wichadakul et al., 2015), *M. anisopliae* (~39.0 Mb; Gao et al., 2011), *M. acridum* (~38.0 Mb; Gao et al., 2011), *C. militaris* (~32.2 Mb; Zheng et al., 2012), *B. bassiana* (Xiao et al., 2012), *T. inflatum* (~30.3 Mb; Bushley et al., 2013), *O. unilateralis* (~23.9 Mb; de Bekker et al., 2017), *O. australis* (~23.3 Mb; de Bekker et al., 2017), and *O. camponoti-rufipedis* (~21.9 Mb; de Bekker et al., 2017) with an exception in *O. sinensis* (Xia et al., 2017) having higher (116.4 Mb) genome size and *H. sinensis* (~102.7 Mb; Jin et al., 2020; Figure 4B). The reason for the higher size in *Hirsutella* and both *Ophiocordyceps* species might be due to the presence of transposable elements (TEs), the chief driving force of genome evolution. *Ophiocordyceps indica* genome harbored 30,441,284 bp of TEs, representing at least 51.38% of the assembly (excluding undefined base Ns). Long terminal repeat (LTR) retrotransposons encompass a total of 18,974,939 bp (~32.03%) in *O. indica* assembly. The GC content was 45.69% across the *O. indica* genome. In comparison, *O. sinensis* has a higher percentage of repeat sequences (74.67%) owing to a rapid increase in the number of TE leading to an increase in genome size (116 Mb), approximately twice the size of the *O. indica* genome. Other insect-fungal pathogens showed a very less number of repeats (~0.98–24.7%) as compared to both *Ophiocordyceps* species (Figure 4C).

Pfam annotations identified 10,718 Pfam domains within 10,613 genes in *O. indica*, which were within the same range (~1% of annotated Pfam) as *O. sinensis* (9,343 domains within 7,939 genes). Furthermore, the numbers of unrepeated Pfam entries were almost similar such as 3,874 (36.5%) for *O. indica* and 3,339 (42.05%) for *O. sinensis*. The most frequent Pfam domains common in both genomes were “WD domain, G-beta repeat,” “Protein kinase domain,” “Major facilitator superfamily,” “Mitochondrial carrier protein,” and “Fungal Zn(2)-Cys(6) binuclear cluster domain.” These observations suggested that they share most of the functions that can determine their lifestyles. The abovementioned Pfam domains represented more than 1% of *O. indica* fungal genome; however, only two Pfam domains (“WD domain, G-beta repeat” and “Protein kinase domain”) were reported to have more than 1% in *O. sinensis*.

In contrast to lower number (7,939) of protein-coding genes (Xia et al., 2017) in *O. sinensis* and *O. camponoti-rufipedis* (7,618; de Bekker et al., 2017), annotated genes of *O. indica* genome were in the expected range (10,613) as in other fungi (average 10,004 genes) *viz*., *M. anisopliae* (10,582), *M. acridum* (9,849), *C. militaris* (9,684), *B. bassiana* (10,366), *O. australis* (8,171), *O. unilateralis* (8,577), *O. polyrhachis-furcata* (10,146), *H. sinensis* (12,058), and *T. inflatum* (9,998). A total of 171 syntenic blocks containing 12,096 collinear genes (~65%) were identified between *O. indica* and *O. sinensis* (Figure 4D), of which ~57% collinear genes of *O. indica* sharing collinearity with ~76% of *O. sinensis*. Furthermore, ~94% non-collinear gene similarity was observed, revealing higher genetic similarity among *Ophiocordyceps* species (Figure 4E). Nearly 2,684 and 496 genes specifically belong to *O. indica* and *O. sinensis*, respectively, wherein 1,918 and 102 proteins were annotated with functional terms based on InterProScan analysis.

Among the specific genes in *O. indica*, the most dominant Gene Ontology terms were associated with oxidation-reduction process (GO: 0055114), transmembrane transport process (GO: 0055085), pathogenesis (GO: 0090729), and DNA-dependent regulation of transcription (GO: 0043565). The finding of oxidation-reduction processes and the trademark for parasite-host interactions including cytochrome P450, FAD, and NAD-binding domains were specific and over-represented in the *O. indica* genome. Other protein-coding genes, *viz*., membrane transporters/branched amino acid metabolism (PF00528/PF02653), Zn (2)-C6 fungal-type (PF00172), LysR bacterial regulatory protein (PF00126), and heat-labile enterotoxin alpha chain domain (PF01375), were most exclusively present.

On the contrary, five genes of a DNA-binding motif (NUMOD3 motif; 2 copies; PF07460) present in the genome of *O. sinensis* were found to be totally absent in the *O. indica* genome. Similarly, a total of 328 syntenic blocks were identified between *O. indica* and *C. militaris* comprising 9,265 collinear genes (~46%) which were also in the same range, considering collinearity genes of *O. sinensis* and *C. militaris*.

### 3.4. Comparison of pathogenesis proteins in *O. indica* fungus with other entomopathogenic fungi

Based on peroxidase profiles, we explored the peroxidase genes amid species of interest using “hmmsearch” program executed in Hmmer (version 3.1b1) package with default parameters. As a result, a total of 54 (0.51%), 42 (0.53%), 36 (0.36%), 28 (0.29%), 36 (0.35%), 42 (0.40%), 41 (0.42%), 28 (0.34%), 40 (0.46%), 37 (0.36%), 34 (0.44%), and 41 (0.22%) peroxidase genes were observed in *O. indica, O. sinensis, T. inflatum, C. militaris, B. bassiana, M. anisopliae, M. acridum, O. australis, O. unilateralis, O. polyrhachis-furcata, O. camponoti-rufipedis*, and *H. sinensis*, respectively. Hidden Markov model searches predicted (~0.51%) peroxidase genes in *O. indica*, remarkably equal to *O. sinensis* (0.53%) and greater than all other fungi analyzed (Figure 4F), proposing that a higher number of peroxidase genes assist in ROS detoxification in both *Ophiocordyceps* species. It is well-reported that secreted enzymes are upregulated during host-pathogen interaction (de Bekker et al., 2017), which includes many pathogenicity-related genes (proteases and heat-labile enterotoxin alpha chain domain). Using MEROPS, a total of 343 (3.23%) protease genes were identified in the *O. indica* (Supplementary Table 2A). In comparison with other fungal species, a comparable number of membrane transport proteins (399) were found in *O. indica* (Supplementary Table 2B). Using the KinBase database, a total of 75 kinases were identified in *O. indica* (Supplementary Table 2C).

The heat-labile enterotoxin alpha chain domain (PF01375) was highly amplified in all entomopathogenic fungi of the family *Ophiocordycipitaceae* except *O. sinensis* (Figure 4F). A large repertoire of 29 (0.27%) heat-labile enterotoxin alpha chain domains was found in the *O. indica* genome in comparison with 9 (0.11%) in the *O. sinensis* (Figure 4F). Enterotoxins are considered important in host-pathogen interactions (Dong and Yao, 2012). Fifteen proteins from a total of 29 encoding heat-labile enterotoxins (alpha chain) were found specific only in the *O. indica* genome and not exclusively shared with *O. sinensis*, reflecting the specificity of the presence of a higher number of enterotoxins, playing an important role in manipulating the behavior of host insects (Tudzynski et al., 2012).

Many insect pathogens require carbohydrate-active enzymes (CAZymes), which are liable to infect their hosts by degrading the cuticle (Cantarel et al., 2009). To detect this, *O. indica* fungus was compared with six other insect pathogens and six plant pathogens. The results revealed that insect pathogens had more proteases and kinases with an exception in the *Ophiocordycipitaceae* family (*O. indica, O. sinensis*, and *T. inflatum*) to degrade the insect cuticle (Ortiz-Urquiza and Keyhani, 2013) as compared with the plant pathogens (Supplementary Table 2). In contrast, plant pathogens possessed more CAZymes than insect pathogens for plant cell wall degradation (Supplementary Table 3). Similar to the other insect pathogens, several cellulase families, including GH7, GH45, and GH51, also declined or were absent in the genome of *O. indica* (Supplementary Table 3A). The detected CAZymes for each fungal species are tabulated in Supplementary Table 3.

### 3.5. Mating system evolution in *O. indica* genome

Genome sequencing analysis depicted that *O. indica* not only possessed the *MAT1-2-1* mating-type gene within the *MAT1-2* idiomorph but also had one mating-type gene (*MAT1-1-3/A-3*) within the *MAT1-1* idiomorph (*MAT* mating-type locus), indicating that *O. indica* fungus is homothallic. The characteristic is extremely different from its closely related heterothallic fungal pathogens, *T. inflatum* (*MAT1-2*), *C. militaris* (*MAT1-1*), *B. bassiana* (*MAT1-1*), *M. anisopliae* (*MAT1-1*), and *M. acridum* (*MAT1-2*), and possess only a single mating-type locus. In conclusion, the homothallic mating behavior observed in both *Ophiocordyceps* species was similar to a well-known homothallic plant pathogen, *F. graminearum* (Kim et al., 2012). To date, *O. sinensis* is reported as the only known homothallic species in *Ophiocordycipitaceae* (Li et al., 2020), and now, we report *O. indica* as the second homothallic species in this family.

### 3.6. Orthology assignment and phylogenetic analysis

The OrthoFinder analysis of 1,95,218 genes from 19 fungal species (*O. indica, O. sinensis, O. australis, O. unilateralis, O. polyrhachis-furcata, O. camponoti-rufipedis, H. sinensis, T. inflatum, M. anisopliae, M. acridum, C. militaris*, and *B. bassiana* as 12 insect-fungal species; *F. graminearum, V. alfalfa, M. grisea, G. clavigera, S. sclerotiorum*, and *B. cinerea* as 6 plant-fungal species; and *Saccharomyces cerevisiae* as outgroup) identified 13,076 gene families/orthologous groups. The total number of genes in the orthologous group was found to be 173,334 (88.8%) while failing to allocate 21,884 (unique) protein-coding genes into any group. One thousand two hundred and thirty-three orthologous groups consisted of a single-copy protein from all fungal species, while an additional 2,234 orthologous groups containing multiple members in one or more species were considered for phylogeny, using RAxML along with *S. cerevisiae* as an outgroup species (Figure 4G). The phylogenetic analysis revealed that the *O. indica* can be considered as the novel species in the family of *Ophiocordycipitaceae* with existing members *O. sinensis, O. unilateralis, O. australis, O. polyrhachis-furcata, O. camponoti-rufipedis, H. sinensis*, and *T. inflatum* (Figure 4H).

### 3.7. Evolution of expanded and contracted gene families in *O. indica*

Computational Analysis of gene Family Evolution (CAFE; Wang and Wang, 2017) identified 117 gene families (with 361 genes) in *O. indica* with significantly higher than expected rate of gains/losses (*P* ≤ 0.05) among 14 fungal species (Figure 4I). *Ophiocordyceps indica* exhibited expansion (significant *P* < 0.01) in gene families, responsible for fungal pathogenicity that comprised of peroxidase (PF01328), heat-labile enterotoxin alpha chain (PF01375), lipase class 3 (PF01764), short-chain dehydrogenase (SDR; PF00106), and N-terminal domain on NACHT_NTPase and P-loop NTPases (PF17107). The highly enriched peroxidase genes were also found to be expanded in *O. sinensis* (Xia et al., 2017); this reflected a strong need for peroxidase genes in both *Ophiocordyceps* species for adapting to the prevailing Himalayan environments. However, those over-represented pathogenic proteins in *O. indica*, such as heat-labile enterotoxin alpha chain and lipase class 3 proteins, were not found to be expanded in *O. sinensis*.

### 3.8. Metabolite profiling

*Ophiocordyceps* species possess different bioactive constituents, *viz*. nucleosides, amino acids, polysaccharides, sterols, proteins, and polypeptides, which make this genus a fungus of medicinal and nutraceutical importance. Therefore, we targeted eight major marker constituents (adenine, adenosine, cordycepin, guanosine, inosine, thymidine, thymine, and uracil) using UPLC-QTOF-MS (Table 5; Supplementary Figures 1–4). The compounds were identified by comparing their retention times and UV spectra with those obtained on injecting standards under the same conditions or by spiking extracts with stock standard solutions. Amazingly, all eight nucleosides were present in *O. indica* and *O. sinensis*. The hot water extraction (2,303.25 and 2,579.83 μg/g) was more efficient than methanolic (80:20) extraction (393.42 and 482.23 μg/g) in *O. indica* and *O. sinensis*, respectively (Table 5). Targeted nucleosides were quantified and confirmed by UV spectra and MS. The total nucleoside content was 1.12 times less in *O. indica*. The most important bioactive marker nucleosides *viz*. cordycepin (59.66 μg/g) and adenosine (243.32 μg/g) showed the product ion at 268.1053 [M + H]+ and 252.1092 [M + H]+, respectively, and were present in fair good quantities in *O. indica;* the corresponding values for these were 21.27 and 627.25 μg/g in *O. sinensis*, respectively. We confirmed cordycepin by ion mobility mass spectrometry technique which was more in *O. indica*. Nucleosides were found in the order of guanosine> inosine> adenosine> adenine> thymidine> uracil> cordycepin> thymine in *O. indica*. A similar trend was observed in *O. sinensis*, except for the quantities of adenosine and inosine (Table 5). In both species, guanosine was found to be the maximum and thymine in the lowest quantities. Thymine was below the quantification level in the hot water extract of *O. sinensis* and was not detected in alcoholic extracts in both *O. indica* and *O. sinensis*.

**Table 5:** Marker nucleosides (μg/g) in *Ophiocordyceps indica* and *O. sinensi*s using different extraction solvents.

**Samples**	**Nucleosides (**μ**g/g) mean** ±**SD**								
	**Uracil**	**Adenine**	**Thymine**	**Inosine**	**Guanosine**	**Thymidine**	**Adenosine**	**Cordycepin**	**Total**
**Hot water extract**									
*O. indica*	67.05 ± 1.14a	190.22 ± 3.61a	8.13 ± 5.94	318.54 ± 17.91a	1,310.71 ± 10.43b	105.17 ± 7.46a	243.32 ± 7.82b	59.66 ± 2.14a	2,303.25 ± 10.70b
*O. sinensis*	25.41 ± 2.00b	152.15 ± 10.69b	0.00 ± 0.00	281.91 ± 26.38a	1,407.77 ± 21.10a	64.07 ± 6.68b	627.25 ± 10.43a	21.27 ± 11.23b	2,579.83 ± 44.40a
**Methanolic (80%) extract**									
*O. indica*	25.50 ± 1.61a	140.88 ± 6.08a	0.00 ± 0.00	7.35 ± 5.28a	101.49 ± 7.26a	49.85 ± 2.62a	52.36 ± 6.71b	15.98 ± 3.18a	393.42 ± 12.68b
*O. sinensis*	28.05 ± 2.74a	127.83 ± 4.17b	0.00 ± 0.00	2.71 ± 2.04b	68.88 ± 5.86b	22.73 ± 16.09b	215.33 ± 11.02a	16.69 ± 2.67a	482.23 ± 22.48a

Values are expressed as mean ± standard deviation (SD; n = 3). The same letters in the same column of each, hot water and methanolic extract are not statistically significant according to Student's t-test with a *P*-value of ≤ 0.001.

A comparable quantity of amino acids (6.15%) was quantified in *O. indica* and is comparable to *O. sinensis* (7.77%). Out of the targeted 14 amino acids, the most abundant were norvaline, arginine, leucine, proline, and valine in *O. indica* (Table 6). Another major component, mannitol, was found higher (10.13%) in *O. indica* as compared to *O. sinensis* (9.06%). Our unpublished results further showed that the toxic heavy metal, arsenic, was not present in *O. indica*. METLIN database analyses revealed the presence of amino acid derivatives, sugars, fatty acids, and nucleosides as the major classes of non-targeted metabolites in the extracts of *O. indica*. The abundance of identified metabolites was visualized using heat maps, illustrating the metabolite variations (Supplementary Figure 5).

**Table 6:** Comparative amino acids in *Ophiocordyceps indica* and *O. sinensis*.

**Samples**	**Amino acids (**μ**g/mg)**															**Total (%)**
	**Ala**	**Arg**	**Asp**	**His**	**Leu**	**Lys**	**Met**	**Nva**	**Pro**	**Ser**	**Thr**	**Trp**	**Tyr**	**Val**	**Total**	
*O. indica*	0.82 ± 0.03b	14.34 ± 0.28b	1.20 ± 0.06a	0.92 ± 0.07a	13.12 ± 0.10b	0.06 ± 0.01b	1.40 ± 0.01b	15.46 ± 0.04a	5.67 ± 0.16b	1.83 ± 0.03b	0.78 ± 0.09a	3.08 ± 0.11a	0.80 ± 0.05b	2.01 ± 0.05b	61.49 ± 5.05b	6.15
*O. sinensis*	1.69 ± 0.06a	14.93 ± 0.24a	1.32 ± 0.10a	0.94 ± 0.04a	13.99 ± 0.09a	4.79 ± 0.07a	3.45 ± 0.01a	14.56 ± 0.10a	6.12 ± 0.16a	2.44 ± 0.08a	3.08 ± 0.08b	2.90 ± 0.04b	4.58 ± 0.10a	2.92 ± 0.08a	77.71 ± 5.56a	7.77

Ala, alanine; Arg, arginine; Asp, asparagine; His, histidine; Leu, leucine; Lys, lysine; Met, methionine; Nva, norvaline; Pro, proline; Ser, serine; Thr, threonine; Trp, tryptophan; Tyr, tyrosine; Val, valine.

Values are expressed as mean ± standard deviation (SD; n = 3). The same letters in the same column are not statistically significant according to Student's t-test with a *P*-value of ≤ 0.001.

### 3.9. Biological activity and safety studies

*Ophiocordyceps indica* exhibited promising antioxidant activity suggesting its therapeutic potential. These activities were statistically at par with *O. sinensis* (Table 7). The IC_50_ DPPH was 1.21 times higher in *O. indica* (2.77 mg/mL) as compared to *O. sinensis* (3.34 mg/mL); however, the reverse trend was observed in IC_50_ ABTS, where *O. sinensis* (0.88 mg/mL) was 1.05 times more efficacious than *O. indica* (0.92 mg/mL). The present findings are in accordance with earlier findings (Singh et al., 2018).

**Table 7:** Comparative antioxidant activity (mg/mL) of *Ophiocordyceps indica* and *O. sinensis*.

**Samples**	**ABTS (mg/mL) IC_50_**	**DPPH (mg/mL) IC_50_**
*O. indica*	0.92 ± 0.31a	2.77 ± 0.72a
*O. sinensis*	0.88 ± 0.37a	3.34 ± 0.99a

Values are expressed as mean ± standard deviation (SD; n = 3). The same letters in the same column are not statistically significant according to Student's t-test with a *P*-value of ≤ 0.05.

**Figure 5:**
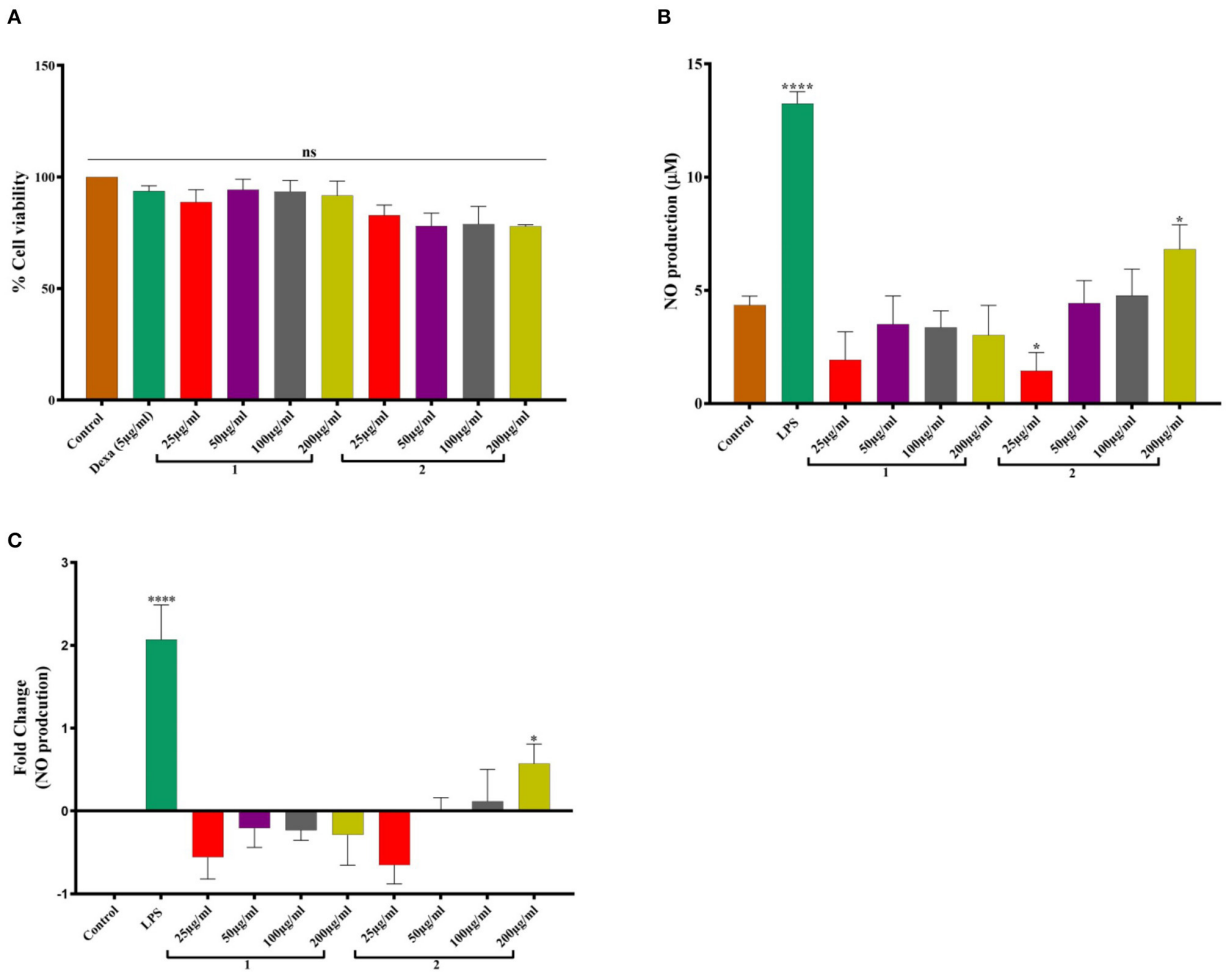
**(A)** Cell viability studies. **(B)** NO production. **(C)** Fold change in NO production in response to 1. Cold water extract and 2. Hot water extract of *O. indica*. Results are expressed as mean ± SD (standard deviation), where *n* = 3. The statistical significance of the data is presented as compared to the control; ns represents that a non-significant difference was observed. * and **** represent statistical significance with *P* < 0.05, and *P* < 0.0001, respectively.

*In vitro* study revealed that hot water extract of *O. indica* was safe and non-toxic and did not compromise cell viability (Figure 5). The extracts of *O. indica* enhanced the nitric oxide (NO) production which is considered responsible for many biological activities including aphrodisiac activities, by virtue of which *O. sinensis* is known as Himalayan Viagra. LPS-stimulated macrophages significantly (*p* ≤ 0.05) enhanced NO production (13.24 ± 0.53 μM) in comparison with control (vehicle control; 4.34 ± 0.34 μM), while hot water extract resulted in higher NO production as compared to cold water extract. These results established the potential of this novel *O. indica* as an activator of NO production.

## 4. Discussion

We reported a novel species, *O. indica*, from the treeline areas (2,202–2,653 m AMSL) of H.P., Indian Western Himalayas. Many *Ophiocordyceps* species have only slight morphological differences; therefore, molecular phylogenetics plays a significant role in confirming their status as separate evolutionary species (Kobmoo et al., 2012; Araújo et al., 2018; Tasanathai et al., 2019). The phylogenetic trees using NJ, ML, and MP methods suggested that *O. indica* is a close relative of *O. xuefengensis, O. sinensis, O. lanpingensis, O. stylophora*, and *H. uncinata*. The multigene analyses also confirmed its distinctiveness among other *Ophiocordyceps* spp. Generated morphological, genetic, chemical, and biological activity data showed its resemblance with the alpine meadow (3,000–5,500 m AMSL) medicinal fungus, *O. sinensis*. Based on habit, habitat, morphology, ITS, multigene (nr*SSU*, nr*LSU, RPB1*, 3′ end *TEF*-*1*α, and *MAT 1-2-1*) phylogenetic, and whole-genome sequence studies, it is identified as a novel species of the genus *Ophiocordyceps*, for which the name *Ophiocordyceps indica* sp. nov. is proposed. Morphologically, this fungus appears somewhat like *O. lanpingensis, O. xuefengensis, O. robertsii*, and *O. stylophora;* however, it is distinct from these species (Table 8). The stroma of *O. indica* is much smaller than *O. xuefengensis*, whereas *O. stylophora* is reported to infect coleopteran larvae and we have recorded it from a lepidopteran larva, *Thitarodes* sp. (Accession No. OR044664). In *O. robertsii*, the fertile part is present at the top of the stroma, whereas in *O. indica* it is present in the middle. *Ophiocordyceps indica* looks quite similar to *O. lanpingensis*, however, it differs in having a hardy stroma as compared to wiry to pliant or fibrous in later (Chen et al., 2013). In the present study, the perithecium of *O. indica* is smaller than *O. robertsii* and *O. sinensis* (Chen et al., 2013). Asci were filiform in *O. indica* as compared to cylindrical in *O. xuefengensis* (Wen et al., 2013). Ascospores were thread-like, multiseptate, hyaline, and filiform (68.0–307.7 × 3.03–5.54 μm) in *O. indica* and are larger in size as compared to filiform ascospores (34.5–48.0 × 2.0–2.4 μm) of *O. acicularis* (Liang, 2007). Filiform, multiseptate ascospores of varying sizes (8.0–470.0 × 1.5–10.0 μm) have been reported in *O. sinensis* (Shrestha et al., 2010). Comparative characteristics of *O*. *indica* and seven phylogenetically closely related species are presented in Table 8, which clearly depicts its distinctiveness from other spp. Thus, the identification and introduction of *O. indica* (a sister taxon of *O. sinensis*), a novel species from low-height areas, are of great significance as it can be an alternative to highly prized *O. sinensis*.

**Table 8:** Comparison of morphological characteristics of *Ophiocordyceps indica* and closely related *Ophiocordyceps* spp.

**Species**	**Host**	**Habitat**	**Perithecium**	**Ascus**	**Ascospore**	**References**
*O. indica*	Lepidoptera larva	Soil	Crowded, oval to flask-shaped, superficial 312–467 × 174–350 μm, loosely packed arranged at right angles to the surface of stroma, followed by tapering part	Cylindrical, filiform, thread-like 150.0–382.2 × 7.58–12.1 μm	Filiform, elongate, thread-like 68.0–307.7 × 3.03–5.54 μm, multiseptate, hyaline, not easy to break into part-spores; part-spores cylindrical	This study
*O. sinensis*	Hepialidae larva	Soil	Nearly superficial, ellipsoidal to ovate, 380– 550 × 140–240 mm	Slender, long, 240–485 × 12–16 μm	2–4 mature ascospores, multiseptate, not breaking into secondary ascospores, 160–470 × 5–6 μm	Liang, 2007
*O. xuefengensis*	Hepialidae larva	Living trunk or upper root near soil	Superficial, long, ovoid, 416–625 × 161–318 μm	Cylindrical, 191–392 × 4.5–8.9 μm, with a 4.2–6.1 μm wide × 3.8–5.8 μm high hemiglobose cap	Thread-like, with many septa, not breaking into secondary ascospores, 130–380 × 1.4–5.2 μm	Wen et al., 2013
*O. lanpingensis*	Hepialidae larva	Grasslands	Oval, 310–370 × 200–240 μm, superficial, oriented at right angles to the surface of the stroma	Cylindrical, 240–300 × 5.1–6.5 μm; with hemispherical ascus cap, 5.4–6.5 μm high, 4.3–5.4 μm wide	Cylindrical, 240–300 × 1.4 μm; not fragments into part-spore, multiseptate with indistinct septation, 3.3–4.9 × 1.1–1.4 μm	Chen et al., 2013
*O. stylophora*	Elateridae larva	Dead wood	Entirely embedded to the surface or at right angles to the surface, narrowly flask- shaped or ovoid, 240–420 × 144–240 μm	Cylindric-clavate, somewhat attenuated below, slightly narrowed above, 170–220 × 8–10 μm	Fusoid-cylindric, 102–164 × 2–3 μm, overlapping in the ascus, multiseptate, the cells 12–29 × 2–3 μm, not breaking into secondary ascospores	Mains, 1941
*O. acicularis*	Elateridae larvae	Soil	Superficial, long ovoid, 360–420 × 200–240 μm	Cylindrical, 7–7.4 μm wide, with a 4.8–5.4 μm wide × 3.6–4.8 μm high hemi- globose cap	Thread-like, multiseptate, 34.5–48 × 2–2.4 μm, not breaking into secondary ascospores	Liang, 2007
*O. robertsii*	Hepialidae larva	Soil	Ascomata superficial, elongate or elliptical, 600–880 × 300– 400 μm	Narrowly cylindrical, 280– 400 × 9–10 μm	Filiform, multiseptate, 280 × 3 μm, breaking into secondary ascospores, 5–6 × 3 μm	Cunningham, 1921
*O. appendiculata*	Coleoptera larva	Soil	Superficial, elliptical, 210– 155 × 130–140 μm	Cylindrical, 50–70 × 7–9 μm	Thread-like, multiseptate (7–8), not breaking into secondary ascospores, 40–63 × 2–2.5 μm	Kobayasi, 1941

*Ophiocordyceps sinensis* is one of the world's most expensive natural medicinal resources, which has and is providing bread and butter for millions of traditional collectors in Himalayan regions (Li Y. et al., 2021; Smith-Hall and Bennike, 2022). Nevertheless, due to habitat degradation, climate change, overexploitation, grazing, and anthropogenic pressures, this fungus has reached an alarming scale of extinction, resulting in it being listed as vulnerable on the IUCN Red List of Threatened Species and the Red List of China's Biodiversity-Macrofungi (Yao et al., 2020; Wei et al., 2021). One of the most common reasons for the reduction in the yield of insect-fungal complex is that the harvesters almost collect all the *Ophiocordyceps* that they encounter, which might result in the production of fewer spores for infecting new caterpillars in the subsequent years. To the best of our knowledge, *O. sinensis* has never been reported from any of the geographical locations, including the alpine meadows and treeline areas of H.P., India, despite resemblances of topography and climatic conditions to that of other locations of its distribution within and outside India. Therefore, the identification of this novel *O. indica* is of tremendous importance and can be exploited for the upliftment of the socio-economic status and livelihood of the rural inhabitants of H.P. through scientific interventions.

If we compare the habitat, *O. indica* is recorded from tree line areas from lower heights, in contrast to the alpine meadow habitat of *O. sinensis*, while the distribution of *O. lanpingensis* is reported from mountain grasses (2,000–3,400 m AMSL) over Hengduan mountain region, China. In the present study, trees (*Picea smithiana, Acer palmatum, Aesculus indica*, and *Prunus cornuta*) and lower plants (*Carpesium* sp., *Diplazium* sp., *Dryopteris* sp., *Impatiens* sp., *Lecanthus* sp., *Pilea* sp., *Potentilla* sp., *Pteris* sp., *Senecio* sp., *Strobilanthes* sp., and *Viola* sp.) were recorded from the sampling areas (Supplementary Videos 1, 2). The soil was covered with fallen tree leaves and decaying organic litter. The soil pH (pH-6.18) of *O. indica* collection site was slightly lower than the pH (pH-5.05–6.07) of soil of Rilkot, Uttarakhand, India, from where *O. sinensis* was collected (Figures 1C, D).

The immature of *O. indica* at the time of collection, as well as after drying, was morphologically quite similar to *O. sinensis* except the color of the caterpillar part and the thickness of the stroma. Caterpillar was little dark brown in *O. indica* as compared to the golden color of *O. sinensis* and the lighter shade of *O. lanpingensis* (Chen et al., 2013). The variation in color from light brown to brown or brownish-black may be attributed to habitat differences as well as melanin concentration due to environmental factors (Dong and Yao, 2012; Shrestha et al., 2012). During the study, we were able to collect a live lepidopteran caterpillar from the soil that was light brownish in color similar to the ghost moth caterpillar, the host of *O. sinensis* as reported in the literature (Figure 1F).

The size, color, weight, stiffness, smell, taste, and robustness of intact caterpillar and stroma decide and influence the demand, market price, and public acceptability of the *Ophiocordyceps* (Zhou et al., 2014; Li Y. et al., 2021), where immature individuals are more valuable (Hopping et al., 2018). Golden-colored and vigorous-size pieces are in great demand in the international market, and generally, 2,000 pieces/kg is considered the best grade (A++) *Ophiocordyceps* (Supplementary Table 4). The best, healthy, and intact specimens of *O. indica* were sorted, weighed, and counted, and excitingly, *O. indica* samples (1,661 pieces/kg) qualify for the best category for fetching high market price.

Our next curiosity and challenge was to find the availability and abundance of *O. indica;* therefore, surveys were conducted in subsequent years and information was gathered from some local inhabitants by showing the collected specimens. Based on the data and information gathered from a few locations only, it is inferred that there is a huge quantity (in Kgs) of *O. indica* in the nearby unexplored areas that was not even collected due to ignorance regarding this highly prized fungus and unavailability of the local market, in addition to fear of administrative rules and regulations for its collection. As of now, there are no guidelines for collection of this novel *O. indica* as it is not reported and recorded earlier. Therefore, it is necessary to frame rules, regulations, and policies, especially for sustainable harvesting and conservation in its natural habitat to avoid overexploitation and overharvesting, as we all witnessed such issues in the case of *O. sinensis*. This can be achieved by declaring some protected areas where harvesting can be totally prohibited or restricted.

*Ophiocordyceps* species are known to be parasitic on many species of insects with intriguing host selectivity. Recently, it was suggested that the parasitic association could have resulted in greater selective pressure to adapt the host's immune response in highly specific niches. The phylogenetic trees suggest that *Ophiocordyceps* species are not a monophyletic group. Furthermore, the analyses of the *O. indica* genome and transcriptome unveiled that gene families associated with fungal pathogenicity have experienced rapid evolution and undergone natural selective pressures. In the present study, of the 22,258 transcripts assembled, ~25,420 (~99.46%) had matches with our genome assembly having parameters >90% coverage and identity. A higher percentage provided by the benchmark approach, BUSCO, in terms of both transcriptome and genomes, validated a high quality of the genome assembly, securing further comparison with entomopathogenic fungi including *O. sinensis*. In contrast to *O. indica*, a global contraction of gene families was apparently observed which evidenced the removal of non-collinear genes and a total loss of gene families in *O. sinensis*.

Insect hosts often expeditiously produce an assload of reactive oxygen species (ROS) against the pathogens, and during evolution, a ROS antioxidant defense system is developed in which peroxidases (ROS-scavenging enzymes) are well-known and fundamental components (Tudzynski et al., 2012). In addition, carbohydrate-active enzymes (CAZymes), responsible for the synthesis, degradation, and modification of all carbohydrates present in the genome including glycoproteins and glycolipids, have also been identified in our study. The functional properties of these gene families revealing expansion status are enriched in the transport process (ABC transporters) and energy metabolism that might have been lost in *O. sinensis* due to accumulation at higher altitudes for the need of conservation of energy. The *O. indica* genome also revealed substantial expansion of gene families that are known to participate in fungal pathogenicity, including peroxidase activity, cytochrome P450, and, especially, heat-labile enterotoxin that was not found to be expanded in the *O. sinensis* genome.

The heat-labile enterotoxin (alpha chain) is known to exhibit manipulation of host insects by interfering with the production of chemical signaling molecules and inferred specificity of the *O. indica* genome. Lipase proteins are involved in the degradation/detoxification of cuticular lipids of insect hosts, thus, inferring their higher importance for pathogenicity (Wang and Wang, 2017). Additionally, these can reduce the energy required to carry out catalytic activity reactions at unusual temperatures. Fungal melanin biosynthesis is dominated by the enzymes involving short-chain dehydrogenase (SDR), which are required for pathogenicity in *M. oryzae* (Wang and Wang, 2017), and thus may play a pivotal role in *O. indica* fungal pathogenesis.

To counter harsh environmental conditions, especially UV radiations, fungi will require UV protectants and heat stabilizers, such as the tyrosinase gene for normal functioning of the cell. A considerable expansion in tyrosinase-enriched gene families (PF00264) in the *O. indica* genome might help in providing an effective way for the stability of spores and resistance to UV radiations (Shang et al., 2012). Membrane transporters are implicated not only in the overall transport of the pathogenic and virulence compounds but also in protection against insect defense compounds as secondary metabolites, probably by exporting the host-derived antimicrobial compounds outside the fungal cell (Merzendorfer, 2014). As expected, highly expanded genes were found to be involved in the transportation process that included ABC transporters (PF00005) and binding-protein-dependent transport system inner membrane component (PF00528) in the genome of *O. indica*. This finding was in contrast to *O. sinensis* where the functional properties of contracted gene families were enriched with transport processes and energy metabolism (Xia et al., 2017).

Some of the enriched PFAM functions were also found related to DNA transposition (Retrotransposon gag protein; PF03732), protein-protein interaction (ankyrin repeats; PF12796), and carbohydrate-binding domain (GLEYA adhesin domain; PF10528). In contrast, gene families exhibiting contraction status (*P* < 0.01) were mainly involved in the pathogenesis such as the WD domain, G-beta repeat (PF00400), and NACHT (PF05729). This finding was in contrast to the expansion of both Pfam domains in *O. sinensis* (Xia et al., 2017); however, the loss of both genes can be thought of compensated by the higher expansion of other pathogenic proteins (heat-labile enterotoxin alpha chain) in the genome of *O. indica*.

In addition, in fungi, mating and sexual development regulators are encoded by mating-type genes. Different sets of mating-type genes are requisite by heterothallic ascomycete species to control non-self-recognition and mating of compatible partners of different mating types. Homothallic (self-fertile) species also carry mating-type genes in their genome that are essential for sexual development (Klix et al., 2010). Generally, the mating system is controlled by the mating-type (*MAT*) locus in Ascomycota fungi (Klix et al., 2010). *MAT1-1-1* and *MAT1-1-3* play a role in fruiting body development, whereas MAT1-1-2 is essential for sexual reproduction (Klix et al., 2010). *O. indica* possessed *MAT1-1* idiomorph including *MAT1-1-3/A-3* mating-type gene and *MAT1-2* idiomorph having *MAT1-2-1* mating-type gene, concluding it to be a homothallic species, the second species of its own type after *O. sinensis* in the family *Ophiocordycipitaceae*.

In addition to identifying any new *Ophiocordyceps* spp., the identification of marker compounds is the utmost requirement for establishing their economic importance. Therefore, metabolomic studies of *O. indica* were conducted and compared with *O. sinensis*. Hot water extraction was the best extraction method and yielded 5.85 times more nucleosides as compared to alcoholic extraction in *O. indica* (Table 5), while it was 5.35 times higher in *O. sinensis*. These results suggest that *O. indica* can be consumed in tea, soup, hot drinks, or other cooked items, just like *O. sinensis* (Chen et al., 2010; Singh et al., 2018; Kaushik et al., 2019). Mannitol, adenosine, and cordycepin are the important qualitative parameters, and our results showed that all these were present in both species. Most of the targeted metabolites were present in both *O. indica* and *O. sinensis*; however, their content varied, possibly due to the difference in metabolism and transformation for nucleosides and amino acids (Chen et al., 2018). Cordycepin is also reported from other species, namely *O. lanpingensis* and *O. xuefengensis* (Chen et al., 2013; Jin et al., 2018). Mannitol was (10.03%) 1.12 times higher in *O. indica* than *O. sinensis* and is almost equal to that reported (10.04%) in natural *O. sinensis* of Sichuan Province, China. Cordycepin content (0.06%) in *O. indica* is the same, while adenosine (0.24%) is 4 times higher than previous records on *O. sinensis* (Zhou et al., 2019). In *O. indica*, adenosine (243.32 μg/g) is higher than in *O. lanpingensis* (113.11 μg/g; Chen et al., 2018). In both *O. indica* and *O. sinensis*, guanosine was maximum as reported earlier in natural and artificial *C. sinensis* (Li et al., 2001). The presence of guanosine has also been reported from *O. lanpingensis* (Chen et al., 2013). Our targeted metabolite approach is further supported by METLIN results, which revealed the presence and matching of 80% of compounds of both species (Supplementary Figure 5). If we look at *O. indica* from a chemical composition point of view (targeted and non-targeted, i.e., METLIN search approach), the presence of a comparable amount of nucleosides, amino acids, mannitol, and other chemical constituents as compared to *O. sinensis* suggests that it can be utilized as an acceptable dietary supplement or pharmacological product after generating necessary safety data.

To the best of our knowledge, this study is the first report of a new species of *Ophiocordyceps* (*O. indica*) from the low-height treeline areas of the Indian Western Himalayan region supported by morphological, whole-genome, and phylogenetic analyses. The results exemplified that *O. indica* is clustered in the genus *Ophiocordyceps* of *Ophiocordycipitaceae* and is closely related to *O. sinensis* and *H. sinensis*. The study presents new insights into the taxonomy, genetics, systematics, and evolution of this important *O. indica*. The comparative analysis with genomes of *Ophiocordyceps* spp. and other fungi will enrich our understanding of the evolution of *Ophiocordycipitaceae*. Furthermore, *O. indica* is identified as the second homothallic species in the family *Ophiocordycipitaceae* after *O. sinensis*.

The identification of this novel *O. indica* from low-height approachable sites will open new avenues for studying biology, host insect-fungus interaction (host screening, breeding, infection rate, and fruiting body development mechanism), cultivation, and other ecological aspects that are difficult to study in other *Ophiocordyceps* spp., especially *O. sinensis* due to its habitat in tough terrains. In addition, many more aspects of this species need to be researched, which will definitely provide new research outcomes. Such in-depth studies will undoubtedly strengthen our findings and lead to further expansion of the subject to exploit *O. indica* as a potential alternative to highly prized medicinal mushrooms, *O. sinensis* “Himalayan Viagra”. These endeavors will help the rural population of the region, by generating employment, thereby improving their livelihood in addition to conserving foreign cash reserves and generating revenue.

## Data availability statement

The datasets presented in this study can be found in online repositories. The names of the repository/repositories and accession number(s) can be found in the article/Supplementary material.

## Ethics statement

In the present study, sample collection was conducted according to India's Biological Diversity Act 2002. This act permits its bonafide citizen to use biological resources for scientific research (Venkataraman, 2009).

## Author contributions

AS: wet lab experiments, data analyses, writing original draft, and manuscript editing. EK: bioinformatics analysis. RJ: chemical profiling and metabolomic studies. PK: experiments. AK: web link and data arrangement on website. DK: chemical profiling, metabolomics, and data analysis. MS: genome sequencing. VA: conceptualization, planning, computational analyses, and writing. GN: conceptualization, planning, execution of the survey, collection of samples, morphological studies, and manuscript editing. All the authors have read, critically reviewed, and approved the submitted version.
