# Supplementary material for: Systematic analyses with genomic and metabolomic insights reveal a new species, Ophiocordyceps indica sp. nov. from treeline area of Indian Western Himalayan region
Source: Front Microbiol. 2023 Jul 20;14:1188649. doi: 10.3389/fmicb.2023.1188649 (PMC10399244; doi:10.3389/fmicb.2023.1188649)

**Supplementary Figures**

**Supplementary Figure 1. UPLC-QTOF-MS and UV of *Ophiocordyceps indica.*** (**A)** PDA standard mixture (Uracil, adenine, thymine, inosine, guanosine, thymidine, adenosine, and cordycepin). (**B**) DAD of *ophiocordyceps indica* extracted in hot water. (**C**) 50% methanol. (**D**) 80% methanol. (**E)** Ethanol. (**F)** Cold water.

**Supplementary Figure 2. TIC and extracted chromatograms.** (**A)** Standard mixture (Uracil, adenine, thymine, inosine, guanosine, thymidine, adenosine, and cordycepin). (**B)** *Ophiocordyceps indica* extracted in hot water. (**C)** 50% methanol. (**D)** 80% methanol. (**E**) Ethanol. (**F**) Cold water.

**Supplementary Figure 3. MS fragmentation pattern of nucleosides.**

**Supplementary Figure 4. ESI and extracted chromatograms** (thymidine, standard mixture, uracil, guanosine, adenosine, cordycepin, inosine, thymine, and adenine).

**Supplementary Figure 5. Heat maps showing differential metabolites in different extracts**. **(A)** Sugars, and derivatives. (**B)** Fatty acids. (**C)** Nucleosides. (**D)** Amino acids and derivatives.

**Supplementary Figure 1. UPLC-QTOF-MS and UV of *Ophiocordyceps indica.*** (**A)** PDA standard mixture (Uracil, adenine, thymine, inosine, guanosine, thymidine, adenosine, and cordycepin). (**B**) DAD of *ophiocordyceps indica* extracted in hot water. (**C**) 50% methanol. (**D**) 80% methanol. (**E)** Ethanol. (**F)** Cold water.

**Supplementary Figure 2. TIC and extracted chromatograms.** (**A)** Standard mixture (Uracil, adenine, thymine, inosine, guanosine, thymidine, adenosine, and cordycepin). (**B)** *Ophiocordyceps indica* extracted in hot water. (**C)** 50% methanol. (**D)** 80% methanol. (**E**) Ethanol. (**F**) Cold water.

**Supplementary Figure 3. MS fragmentation pattern of nucleosides.**

**Supplementary Figure 4. ESI and extracted chromatograms** (thymidine, standard mixture, uracil, guanosine, adenosine, cordycepin, inosine, thymine, and adenine).

**Supplementary Figure 5. Heat maps showing differential metabolites in different extracts**. **(A)** Sugars, and derivatives. (**B)** Fatty acids. (**C)** Nucleosides. (**D)** Amino acids and derivatives.


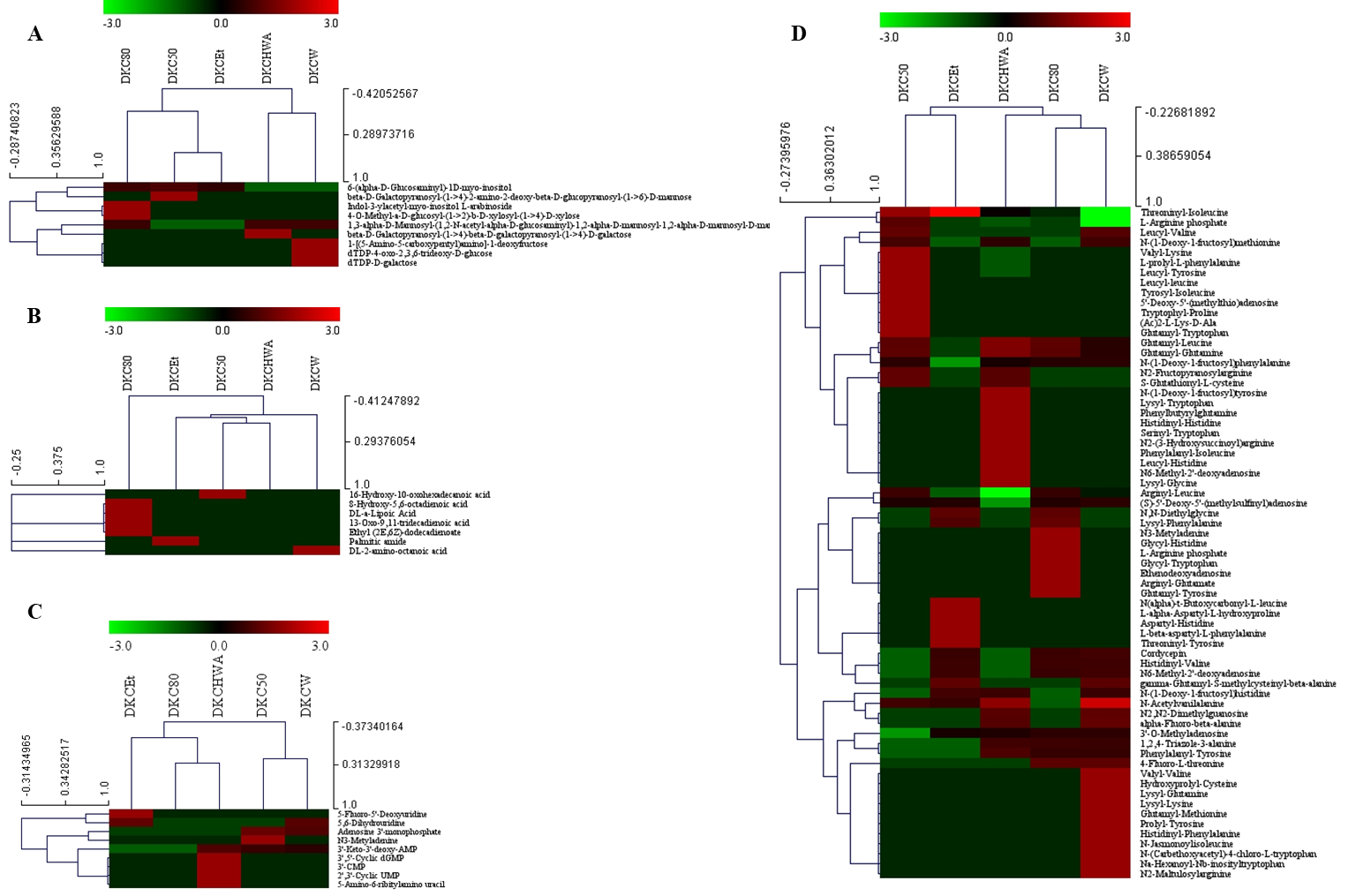

Supplement: Supplementary file 1 [file Data_Sheet_1.docx]
