# Supplementary material for: Systematic analyses with genomic and metabolomic insights reveal a new species, Ophiocordyceps indica sp. nov. from treeline area of Indian Western Himalayan region
Source: Front Microbiol. 2023 Jul 20;14:1188649. doi: 10.3389/fmicb.2023.1188649 (PMC10399244; doi:10.3389/fmicb.2023.1188649)
Supplement: Supplementary file 2 [file Table_1.docx]

**Supplementary Tables**

**Supplementary Table 1(a-c)** Data for taxa used in the sequence analysis.

**Table 1a**

| **Species** | **Gene Bank No.** | **Species** | **Gene Bank No.** |
| --- | --- | --- | --- |
| ***Beauveria bassiana*** | NR111594 | ***O. robertsii*** | KC167175 |
| ***Cordyceps emeiensis*** | AJ309347 | ***O. robertsii*** | JX177490 |
| ***C. morakotii*** | NR147575 | ***O. stylophora*** | LC042504 |
| ***C. robertsii*** | AJ309335 | ***O. arborescens*** | NR147510 |
| ***C. sinensis*** | AJ243776 | ***O. retorta*** | NR163285 |
| ***Cordyceps sp.*** | EF495106 | ***O. xuefengensis*** | KC631803 |
| ***C. yinjiangensis*** | NR171876 | ***O. xuefengensis*** | NR155561 |
| ***Hirsutella illustris*** | KM652160 | ***O. macroacicularis*** | AB968400 |
| ***H. liboensis*** | NR166545 | ***O. appendiculata*** | JN943325 |
| ***H. rhossiliensis*** | DQ345575 | ***O. lanpingensis*** | HQ654775 |
| ***H. rhossiliensis*** | DQ345586 | ***O. sinensis*** | KX082966 |
| ***H. rhossiliensis*** | NR145063 | ***O. lanpingensis*** | HQ654776 |
| ***Hirsutella sp.*** | JF320817 | ***O. stylophora*** | JN049828 |
| ***H. uncinata*** | KJ524691 | ***O. longissimi*** | MG031298 |
| ***H. uncinata*** | NR111154 | ***O. macroacicularis*** | NR14751 |
| ***H. vermicola*** | NR137547 | ***O. sinensis*** | KX082968 |
| ***Metacordyceps liangshanensis*** | KJ021169 | ***O. macroacicularis*** | KR005829 |
| ***M. liangshanensis*** | KJ021170 | ***Tolypocladium album*** | NR155018 |
| ***Ophiocordyceps indica*** | KY486751 | ***T. geodes*** | NR164431 |
| ***O.***  ***robertsii*** | KC167174 | ***T. inflatum*** | NR171737 |
| ***Ophiocordyceps sp.*** | HQ654777 | ***T. inusitaticapitatum*** | NR175695 |
| ***O. xuefengensis*** | KC631804 | ***T. ovalisporum*** | NR155019 |
| ***O. appendiculata*** | JN943326 | ***T. tropicale*** | NR159005 |
| ***O. sobolifera*** | MT349957 |  |  |

**Table 1b**

| **Species** | **Specimen voucher** | **Gene Bank Accession No.** | | | |
| --- | --- | --- | --- | --- | --- |
|  |  | **nr*SSU*** | **nr*LSU*** | ***tef-1*** | ***rpb1*** |
| ***Cordyceps unilateralis*** | OSC 128574 | [DQ522554](http://www.ncbi.nlm.nih.gov/entrez/query.fcgi?cmd=search&db=nucleotide&doptcmdl=genbank&term=DQ522554) | [DQ518768](http://www.ncbi.nlm.nih.gov/entrez/query.fcgi?cmd=search&db=nucleotide&doptcmdl=genbank&term=DQ518768) | [DQ522339](http://www.ncbi.nlm.nih.gov/entrez/query.fcgi?cmd=search&db=nucleotide&doptcmdl=genbank&term=DQ522339) | [DQ522385](http://www.ncbi.nlm.nih.gov/entrez/query.fcgi?cmd=search&db=nucleotide&doptcmdl=genbank&term=DQ522385) |
| ***Cordyceps sp.*** | OSC 110997 | [EF468976](http://www.ncbi.nlm.nih.gov/entrez/query.fcgi?cmd=search&db=nucleotide&doptcmdl=genbank&term=EF468976) | Data missing | [EF468774](http://www.ncbi.nlm.nih.gov/entrez/query.fcgi?cmd=search&db=nucleotide&doptcmdl=genbank&term=EF468774) | [EF468879](http://www.ncbi.nlm.nih.gov/entrez/query.fcgi?cmd=search&db=nucleotide&doptcmdl=genbank&term=EF468879) |
| ***Hirsutella rhossiliensis*** | CBS 211.77 | MH861052 | MH872820 | KP886321 | Data missing |
| ***H. rhossiliensis*** | ARSEF3751 | KM652081 | KM652124 | KM652007 | KM652046 |
| ***Hirsutella sp.*** | NHJ 12525 | [EF469125](http://www.ncbi.nlm.nih.gov/entrez/query.fcgi?cmd=search&db=nucleotide&doptcmdl=genbank&term=EF469125) | [EF469078](http://www.ncbi.nlm.nih.gov/entrez/query.fcgi?cmd=search&db=nucleotide&doptcmdl=genbank&term=EF469078) | [EF469063](http://www.ncbi.nlm.nih.gov/entrez/query.fcgi?cmd=search&db=nucleotide&doptcmdl=genbank&term=EF469063) | [EF469092](http://www.ncbi.nlm.nih.gov/entrez/query.fcgi?cmd=search&db=nucleotide&doptcmdl=genbank&term=EF469092) |
| ***Ophiocordyceps acicularis*** | OSC 110987 | [EF468950](http://www.ncbi.nlm.nih.gov/entrez/query.fcgi?cmd=search&db=nucleotide&doptcmdl=genbank&term=EF468950) | [EF468805](http://www.ncbi.nlm.nih.gov/entrez/query.fcgi?cmd=search&db=nucleotide&doptcmdl=genbank&term=EF468805) | [EF468744](http://www.ncbi.nlm.nih.gov/entrez/query.fcgi?cmd=search&db=nucleotide&doptcmdl=genbank&term=EF468744) | [EF468852](http://www.ncbi.nlm.nih.gov/entrez/query.fcgi?cmd=search&db=nucleotide&doptcmdl=genbank&term=EF468852) |
| ***O. acicularis*** | OSC 110988 | [EF468951](http://www.ncbi.nlm.nih.gov/entrez/query.fcgi?cmd=search&db=nucleotide&doptcmdl=genbank&term=EF468951) | [EF468804](http://www.ncbi.nlm.nih.gov/entrez/query.fcgi?cmd=search&db=nucleotide&doptcmdl=genbank&term=EF468804) | [EF468745](http://www.ncbi.nlm.nih.gov/entrez/query.fcgi?cmd=search&db=nucleotide&doptcmdl=genbank&term=EF468745) | [EF468853](http://www.ncbi.nlm.nih.gov/entrez/query.fcgi?cmd=search&db=nucleotide&doptcmdl=genbank&term=EF468853) |
| ***O. acicularis*** | OSC 128580 | [DQ522543](http://www.ncbi.nlm.nih.gov/entrez/query.fcgi?cmd=search&db=nucleotide&doptcmdl=genbank&term=DQ522543) | [DQ518757](http://www.ncbi.nlm.nih.gov/entrez/query.fcgi?cmd=search&db=nucleotide&doptcmdl=genbank&term=DQ518757) | [DQ522326](http://www.ncbi.nlm.nih.gov/entrez/query.fcgi?cmd=search&db=nucleotide&doptcmdl=genbank&term=DQ522326) | [DQ522371](http://www.ncbi.nlm.nih.gov/entrez/query.fcgi?cmd=search&db=nucleotide&doptcmdl=genbank&term=DQ522371) |
| ***O. agriota*** | ARSEF 5692 | [DQ522540](http://www.ncbi.nlm.nih.gov/entrez/query.fcgi?cmd=search&db=nucleotide&doptcmdl=genbank&term=DQ522540) | [DQ518754](http://www.ncbi.nlm.nih.gov/entrez/query.fcgi?cmd=search&db=nucleotide&doptcmdl=genbank&term=DQ518754) | [DQ522322](http://www.ncbi.nlm.nih.gov/entrez/query.fcgi?cmd=search&db=nucleotide&doptcmdl=genbank&term=DQ522322) | [DQ522368](http://www.ncbi.nlm.nih.gov/entrez/query.fcgi?cmd=search&db=nucleotide&doptcmdl=genbank&term=DQ522368) |
| ***O. agriotidis*** | ARSEF 5692 | DQ522540 | DQ518754 | DQ522322 | DQ522368 |
| ***O. aphodii*** | ARSEF 5498 | DQ522541 | DQ518755 | DQ522323 | Data missing |
| ***O. appendiculata*** | NBRC 106959 | JN941729 | JN941412 | AB968578 | JN992463 |
| ***O. appendiculata*** | NBRC 106960 | JN941728 | JN941413 | AB968577 | JN992462 |
| ***O. arborescens*** | NBRC105891 | NG064858 | NG060238 | AB968572 | Data missing |
| ***O. brunneipunctata*** | OSC 128576 | DQ522542 | DQ518756 | DQ522324 | DQ522369 |
| ***O. clavata*** | NBRC 106961 | JN941727 | JN941414 | AB968586 | JN992461 |
| ***O. clavata*** | NBRC 106962 | JN941726 | JN941415 | AB968587 | JN992460 |
| ***O. cuboidea*** | NBRC 100941 | JN941725 | JN941416 | Data missing | JN992459 |
| ***O. entomorrhiza*** | KEW 53484 | EF468954 | EF468809 | EF468749 | EF468857 |
| ***O. gracilis*** | EFCC 3101 | EF468955 | EF468810 | EF468750 | EF468858 |
| ***O. gracilis*** | EFCC 8572 | EF468956 | EF468811 | EF468751 | EF468859 |
| ***O. heteropoda*** | EFCC 10125 | EF468957 | EF468812 | EF468752 | EF468860 |
| ***O. indica*** |  | MZ571406 | KY486751 | MZ514914 | MZ514912 |
| ***O. irangiensis*** | OSC 128577 | DQ522546 | DQ518760 | DQ522329 | DQ522374 |
| ***O. irangiensis*** | OSC 128579 | EF469123 | EF469076 | EF469060 | EF469089 |
| ***O. lanpingensis*** | YHOS0707 | KC417459 | KC417461 | KC417463 | KC417465 |
| ***O. lanpingensis*** | YHOS0705 | KC417458 | KC417460 | KC417462 | KC417464 |
| ***O. macroasicularis*** | NRBC100685 | NG064859 | NG060239 | AB968574 | KC1799 |
| ***O. melolonthae*** | OSC 110993 | DQ522548 | DQ518762 | DQ522331 | DQ522376 |
| ***O. nutans*** | NBRC101749 | JN941712 | JN491429 | AB968589 | AB968589 |
| ***O. ravenelii*** | OSC 110995 | DQ522550 | DQ522550 | DQ522334 | DQ522379 |
| ***O. rhizoidea*** | NHJ 12522 | EF468970 | EF468825 | EF468764 | EF468873 |
| ***O. rhizoidea*** | NHJ 12529 | EF468969 | EF468824 | EF468765 | EF468872 |
| ***O. robertsii*** | YHORZT007 | KC561978 | KC561978 | KC561979 | KC561980 |
| ***O. rubiginosiperitheciata*** | NBRC 100946 | JN941705 | JN941436 | AB968581 | JN992439 |
| ***O. rubiginosiperitheciata*** | NBRC 106966 | JN941704 | JN941437 | AB968582 | JN992438 |
| ***O. sinensis*** | SJL0809 | [HM135169](http://www.ncbi.nlm.nih.gov/entrez/query.fcgi?cmd=search&db=nucleotide&doptcmdl=genbank&term=HM135169) | [HM135168](http://www.ncbi.nlm.nih.gov/entrez/query.fcgi?cmd=search&db=nucleotide&doptcmdl=genbank&term=HM135168) | [HM140637](http://www.ncbi.nlm.nih.gov/entrez/query.fcgi?cmd=search&db=nucleotide&doptcmdl=genbank&term=HM140637) | [HM140640](http://www.ncbi.nlm.nih.gov/entrez/query.fcgi?cmd=search&db=nucleotide&doptcmdl=genbank&term=HM140640) |
| ***O. sinensis*** | EFCC 7287 | [EF468971](http://www.ncbi.nlm.nih.gov/entrez/query.fcgi?cmd=search&db=nucleotide&doptcmdl=genbank&term=EF468971) | [EF468827](http://www.ncbi.nlm.nih.gov/entrez/query.fcgi?cmd=search&db=nucleotide&doptcmdl=genbank&term=EF468827) | [EF468767](http://www.ncbi.nlm.nih.gov/entrez/query.fcgi?cmd=search&db=nucleotide&doptcmdl=genbank&term=EF468767) | [EF468874](http://www.ncbi.nlm.nih.gov/entrez/query.fcgi?cmd=search&db=nucleotide&doptcmdl=genbank&term=EF468874) |
| ***O. sphecocephala*** | OSC 110998 | DQ522551 | DQ518765 | DQ522336 | DQ522381 |
| ***O. stylophora*** | OSC 111000 | [DQ522552](http://www.ncbi.nlm.nih.gov/entrez/query.fcgi?cmd=search&db=nucleotide&doptcmdl=genbank&term=DQ522552) | [DQ518766](http://www.ncbi.nlm.nih.gov/entrez/query.fcgi?cmd=search&db=nucleotide&doptcmdl=genbank&term=DQ518766) | [DQ522337](http://www.ncbi.nlm.nih.gov/entrez/query.fcgi?cmd=search&db=nucleotide&doptcmdl=genbank&term=DQ522337) | [DQ522382](http://www.ncbi.nlm.nih.gov/entrez/query.fcgi?cmd=search&db=nucleotide&doptcmdl=genbank&term=DQ522382) |
| ***O. stylophora*** | OSC 111000 | DQ522552 | DQ518766 | DQ522337 | DQ522382 |
| ***O. variabilis*** | ARSEF 5365 | DQ522555 | DQ518769 | DQ522340 | DQ522386 |
| ***O. variabilis*** | OSC 111003 | EF468985 | EF468839 | EF468779 | EF468885 |
| ***O. xuefengensis*** | GZUH2012HN14 | NG065010 | Data missing | KC631793 | KC631798 |
| ***O. xuefengensis*** | GZUH2012HN19 | KC631803 | Data missing | KC631794 | Data missing |
| ***O. xuefengensis*** | GZUH2012HN13 | KC631787 | Data missing | KC631792 | KC631797 |
| ***Tolypocladium inflatum*** | OSB71235 | EF469124 | EF469077 | EF469061 | EF469090 |

**Table 1c**

| **Species** | **Gene Bank No.** | **Species** | **Gene Bank No.** |
| --- | --- | --- | --- |
| ***Cordyceps bassiana*** | AB124625 | ***O. robertsii*** | KC561976 |
| ***C. brongniartii*** | AB258379 | ***O. sinensis*** | KC429556 |
| ***C. chlamydosporia*** | AB255627 | ***O. sinensis*** | KC429555 |
| ***C. cicadae*** | AB124628 | ***O. sinensis*** | KC429554 |
| ***C. confragosa*** | AB124636 | ***O. sinensis*** | KC429550 |
| ***C. cylindrica*** | AB255620 | ***O. sinensis*** | FJ654178 |
| ***C. militaris*** | AB124626 | ***O. sinensis*** | KC429557 |
| ***Ophiocordyceps indica*** | MZ514913 | ***O. xuefengensis*** | MH176301 |
| ***O. lanpingensis*** | KC561977 |  |  |

**Supplementary Table S3a.** Distribution of Carbohydrate-degrading enzymes among *Ophiocordyceps indica* and other twelve fungal genomes arranged by Glycoside Hydrolases (GH) gene family.

| **GH**  **FAMILY** | **OIN** | **OSI** | **MAN** | **MAC** | **CMI** | **BBA** | **TOL** | **FGR** | **MGR** | **GCL** | **SSC** | **BCI** | **VER** |
| --- | --- | --- | --- | --- | --- | --- | --- | --- | --- | --- | --- | --- | --- |
| GH1 | 2 | 1 | 3 | 3 | 1 | 1 | 3 | 3 | 2 | 1 | 3 | 2 | 4 |
| GH2 | 4 | 3 | 5 | 6 | 6 | 7 | 4 | 10 | 8 | 3 | 2 | 2 | 7 |
| GH3 | 7 | 6 | 7 | 7 | 9 | 11 | 7 | 21 | 18 | 14 | 13 | 15 | 21 |
| GH5 | 4 | 3 | 8 | 9 | 7 | 16 | 6 | 14 | 13 | 9 | 14 | 16 | 14 |
| GH6 | 1 | 1 | 0 | 0 | 0 | 0 | 0 | 1 | 3 | 0 | 1 | 1 | 4 |
| GH7 | 0 | 0 | 0 | 0 | 0 | 0 | 0 | 2 | 5 | 4 | 3 | 2 | 6 |
| GH10 | 0 | 0 | 0 | 0 | 0 | 0 | 0 | 5 | 6 | 0 | 2 | 2 | 4 |
| GH11 | 0 | 0 | 0 | 0 | 0 | 0 | 0 | 3 | 5 | 1 | 3 | 3 | 4 |
| GH12 | 1 | 1 | 1 | 1 | 1 | 1 | 1 | 4 | 4 | 5 | 4 | 5 | 5 |
| GH13 | 5 | 4 | 6 | 5 | 5 | 5 | 5 | 8 | 10 | 5 | 12 | 13 | 6 |
| GH15 | 1 | 1 | 2 | 2 | 2 | 2 | 2 | 3 | 2 | 2 | 4 | 5 | 4 |
| GH16 | 16 | 13 | 20 | 18 | 20 | 24 | 19 | 23 | 18 | 11 | 20 | 21 | 12 |
| GH17 | 6 | 5 | 6 | 4 | 4 | 4 | 5 | 4 | 7 | 3 | 6 | 5 | 6 |
| GH18 | 16 | 15 | 25 | 18 | 22 | 18 | 17 | 18 | 16 | 10 | 14 | 11 | 13 |
| GH20 | 1 | 2 | 2 | 2 | 3 | 4 | 2 | 3 | 3 | 2 | 1 | 1 | 2 |
| GH23 | 1 | 0 | 1 | 0 | 1 | 0 | 1 | 0 | 0 | 0 | 0 | 0 | 0 |
| GH24 | 0 | 0 | 2 | 1 | 0 | 0 | 0 | 0 | 0 | 0 | 0 | 0 | 1 |
| GH25 | 0 | 0 | 2 | 2 | 1 | 1 | 1 | 0 | 0 | 0 | 1 | 1 | 0 |
| GH26 | 0 | 0 | 0 | 0 | 0 | 0 | 0 | 0 | 0 | 0 | 1 | 2 | 1 |
| GH27 | 0 | 1 | 2 | 2 | 0 | 2 | 3 | 2 | 3 | 0 | 4 | 4 | 3 |
| GH28 | 1 | 1 | 1 | 1 | 1 | 1 | 1 | 6 | 4 | 5 | 17 | 17 | 13 |
| GH29 | 0 | 0 | 1 | 2 | 2 | 2 | 0 | 1 | 4 | 0 | 0 | 0 | 0 |
| GH30 | 0 | 0 | 0 | 0 | 0 | 0 | 1 | 0 | 1 | 0 | 0 | 0 | 1 |
| GH31 | 2 | 2 | 6 | 6 | 4 | 6 | 4 | 8 | 6 | 1 | 6 | 4 | 8 |
| GH32 | 0 | 1 | 1 | 2 | 1 | 1 | 2 | 6 | 5 | 0 | 1 | 1 | 3 |
| GH33 | 0 | 0 | 1 | 1 | 1 | 0 | 0 | 1 | 1 | 0 | 0 | 0 | 2 |
| GH35 | 1 | 1 | 3 | 3 | 3 | 3 | 1 | 3 | 0 | 1 | 4 | 4 | 5 |
| GH36 | 1 | 1 | 1 | 1 | 1 | 1 | 0 | 2 | 0 | 0 | 0 | 0 | 1 |
| GH37 | 2 | 1 | 2 | 2 | 2 | 2 | 2 | 2 | 2 | 2 | 1 | 1 | 2 |
| GH38 | 1 | 1 | 1 | 1 | 1 | 1 | 1 | 1 | 2 | 0 | 1 | 1 | 2 |
| GH39 | 0 | 0 | 0 | 0 | 0 | 0 | 0 | 0 | 1 | 0 | 0 | 0 | 1 |
| GH43 | 2 | 1 | 1 | 0 | 0 | 1 | 0 | 16 | 17 | 8 | 5 | 5 | 22 |
| GH45 | 0 | 0 | 0 | 0 | 0 | 1 | 0 | 1 | 1 | 1 | 2 | 2 | 1 |
| GH47 | 7 | 7 | 8 | 9 | 7 | 7 | 7 | 10 | 9 | 6 | 8 | 7 | 8 |
| GH51 | 0 | 0 | 0 | 0 | 0 | 0 | 0 | 2 | 3 | 2 | 2 | 1 | 2 |
| GH53 | 0 | 0 | 0 | 0 | 0 | 0 | 0 | 1 | 1 | 1 | 2 | 2 | 1 |
| GH54 | 0 | 0 | 1 | 0 | 1 | 1 | 0 | 1 | 1 | 0 | 1 | 1 | 1 |
| GH55 | 2 | 2 | 4 | 4 | 8 | 7 | 4 | 3 | 4 | 3 | 4 | 4 | 3 |
| GH62 | 0 | 0 | 0 | 0 | 0 | 0 | 0 | 2 | 4 | 0 | 0 | 1 | 0 |
| GH63 | 1 | 1 | 1 | 1 | 2 | 2 | 1 | 1 | 1 | 1 | 1 | 1 | 0 |
| GH64 | 2 | 3 | 1 | 1 | 2 | 1 | 2 | 2 | 2 | 1 | 2 | 2 | 2 |
| GH65 | 0 | 0 | 1 | 1 | 1 | 0 | 0 | 0 | 0 | 0 | 3 | 2 | 0 |
| GH67 | 0 | 0 | 0 | 0 | 0 | 0 | 0 | 1 | 1 | 0 | 0 | 0 | 1 |
| GH71 | 0 | 0 | 1 | 1 | 0 | 0 | 1 | 0 | 1 | 0 | 8 | 10 | 2 |
| GH72 | 5 | 6 | 5 | 5 | 5 | 5 | 5 | 3 | 5 | 2 | 6 | 6 | 4 |
| GH74 | 2 | 2 | 2 | 2 | 0 | 0 | 3 | 4 | 5 | 2 | 2 | 3 | 8 |
| GH75 | 1 | 1 | 3 | 3 | 2 | 2 | 1 | 1 | 1 | 0 | 0 | 0 | 1 |
| GH76 | 8 | 8 | 14 | 13 | 10 | 8 | 6 | 8 | 8 | 7 | 12 | 12 | 7 |
| GH78 | 0 | 0 | 0 | 0 | 0 | 0 | 1 | 7 | 4 | 5 | 4 | 8 | 10 |
| GH79 | 0 | 0 | 2 | 1 | 1 | 2 | 3 | 1 | 2 | 0 | 2 | 2 | 2 |
| GH81 | 1 | 1 | 1 | 2 | 1 | 1 | 1 | 1 | 3 | 1 | 1 | 1 | 3 |
| GH84 | 0 | 0 | 1 | 1 | 1 | 1 | 0 | 0 | 0 | 0 | 0 | 0 | 0 |
| GH88 | 0 | 0 | 1 | 0 | 1 | 1 | 1 | 1 | 1 | 0 | 0 | 1 | 4 |
| GH89 | 1 | 0 | 2 | 1 | 2 | 2 | 1 | 0 | 0 | 0 | 1 | 0 | 0 |
| GH92 | 3 | 3 | 5 | 4 | 4 | 4 | 4 | 0 | 6 | 3 | 5 | 5 | 3 |
| GH93 | 0 | 0 | 0 | 0 | 0 | 0 | 1 | 2 | 1 | 0 | 1 | 1 | 1 |
| GH94 | 0 | 0 | 0 | 0 | 0 | 0 | 0 | 0 | 1 | 0 | 0 | 0 | 0 |
| GH95 | 0 | 0 | 1 | 0 | 0 | 1 | 0 | 2 | 1 | 0 | 2 | 3 | 3 |
| GH99 | 0 | 0 | 1 | 0 | 0 | 0 | 0 | 0 | 0 | 0 | 0 | 0 | 0 |
| GH105 | 1 | 1 | 3 | 1 | 0 | 0 | 1 | 3 | 3 | 1 | 1 | 1 | 4 |
| GH106 | 0 | 0 | 0 | 0 | 0 | 0 | 0 | 1 | 1 | 1 | 1 | 1 | 1 |
| GH109 | 3 | 3 | 7 | 7 | 6 | 8 | 7 | 15 | 8 | 4 | 5 | 5 | 13 |
| GH114 | 1 | 0 | 1 | 1 | 2 | 1 | 2 | 2 | 1 | 0 | 1 | 1 | 2 |
| GH115 | 0 | 0 | 1 | 1 | 0 | 0 | 1 | 3 | 4 | 0 | 1 | 2 | 3 |
| GH117 | 0 | 0 | 1 | 0 | 0 | 0 | 0 | 0 | 0 | 0 | 0 | 0 | 0 |
| GH121 | 0 | 0 | 0 | 0 | 0 | 0 | 0 | 0 | 0 | 0 | 0 | 0 | 0 |
| GH125 | 2 | 2 | 3 | 3 | 2 | 3 | 4 | 3 | 4 | 3 | 3 | 3 | 1 |
| GH127 | 1 | 1 | 1 | 1 | 1 | 1 | 1 | 2 | 2 | 2 | 0 | 1 | 1 |
| GH128 | 2 | 2 | 5 | 3 | 2 | 3 | 2 | 4 | 4 | 2 | 3 | 4 | 3 |
| GH131 | 0 | 0 | 0 | 0 | 0 | 0 | 0 | 1 | 7 | 1 | 2 | 1 | 2 |
| GH132 | 2 | 2 | 2 | 2 | 2 | 2 | 3 | 2 | 2 | 2 | 2 | 2 | 2 |
| **TOTAL** | 120 | 110 | 189 | 167 | 161 | 178 | 151 | 260 | 268 | 138 | 231 | 240 | 276 |

**Supplementary Table S3b.** Distribution of Carbohydrate-degrading enzymes among *Ophiocordyceps indica* and other twelve fungal genomes arranged by Carbohydrate-Binding Modules (CBM) gene family.

| **CBM FAMILY** | **OIN** | **OSI** | **MAN** | **MAC** | **CMI** | **BBA** | **TOL** | **FGR** | **MGR** | **GCL** | **SSC** | **BCI** | **VER** |
| --- | --- | --- | --- | --- | --- | --- | --- | --- | --- | --- | --- | --- | --- |
| CBM1 | 0 | 0 | 3 | 2 | 1 | 3 | 3 | 13 | 25 | 2 | 21 | 17 | 22 |
| CBM6 | 0 | 0 | 0 | 0 | 0 | 0 | 0 | 1 | 2 | 0 | 0 | 0 | 0 |
| CBM9 | 0 | 0 | 0 | 1 | 0 | 0 | 0 | 0 | 0 | 0 | 0 | 0 | 0 |
| CBM13 | 1 | 1 | 1 | 2 | 4 | 5 | 1 | 3 | 2 | 1 | 4 | 3 | 1 |
| CBM16 | 0 | 0 | 0 | 0 | 0 | 0 | 0 | 4 | 0 | 0 | 0 | 0 | 0 |
| CBM18 | 8 | 4 | 5 | 2 | 11 | 8 | 4 | 29 | 54 | 5 | 24 | 12 | 17 |
| CBM19 | 0 | 0 | 0 | 0 | 0 | 1 | 0 | 0 | 0 | 0 | 0 | 0 | 0 |
| CBM20 | 1 | 4 | 1 | 1 | 1 | 1 | 1 | 2 | 2 | 2 | 4 | 3 | 4 |
| CBM21 | 1 | 1 | 3 | 2 | 1 | 1 | 1 | 2 | 1 | 1 | 1 | 1 | 1 |
| CBM22 | 0 | 0 | 0 | 0 | 0 | 0 | 0 | 3 | 0 | 0 | 0 | 0 | 0 |
| CBM24 | 1 | 1 | 2 | 1 | 0 | 0 | 1 | 2 | 0 | 0 | 12 | 13 | 4 |
| CBM32 | 0 | 0 | 0 | 0 | 0 | 0 | 0 | 0 | 1 | 1 | 0 | 0 | 0 |
| CBM35 | 0 | 0 | 0 | 0 | 0 | 0 | 0 | 2 | 2 | 0 | 1 | 2 | 3 |
| CBM40 | 0 | 0 | 0 | 0 | 0 | 0 | 0 | 0 | 1 | 0 | 0 | 0 | 0 |
| CBM42 | 0 | 0 | 1 | 0 | 1 | 1 | 0 | 1 | 2 | 0 | 1 | 1 | 2 |
| CBM43 | 2 | 2 | 2 | 2 | 2 | 2 | 2 | 1 | 2 | 1 | 1 | 1 | 1 |
| CBM46 | 0 | 0 | 0 | 0 | 0 | 0 | 0 | 0 | 0 | 0 | 1 | 1 | 1 |
| CBM48 | 1 | 1 | 1 | 1 | 1 | 1 | 1 | 1 | 1 | 1 | 1 | 1 | 1 |
| CBM50 | 13 | 14 | 12 | 3 | 17 | 15 | 9 | 12 | 11 | 14 | 7 | 1 | 11 |
| CBM52 | 0 | 0 | 0 | 0 | 0 | 1 | 0 | 0 | 1 | 1 | 0 | 0 | 0 |
| CBM61 | 0 | 0 | 0 | 0 | 0 | 0 | 0 | 2 | 0 | 0 | 2 | 1 | 0 |
| CBM63 | 0 | 0 | 0 | 0 | 0 | 0 | 0 | 3 | 1 | 1 | 1 | 1 | 1 |
| CBM66 | 1 | 0 | 3 | 3 | 4 | 1 | 7 | 0 | 1 | 0 | 0 | 1 | 0 |
| CBM67 | 0 | 0 | 0 | 0 | 0 | 0 | 0 | 4 | 1 | 1 | 1 | 3 | 6 |
| **TOTAL** | **29** | **28** | **34** | **20** | **43** | **40** | **30** | **84** | **108** | **31** | **82** | **62** | **75** |

**Supplementary Table S3c.** Distribution of Carbohydrate-degrading enzymes among *Ophiocordyceps indica* and other twelve fungal genomes arranged by Carbohydrate Esterases (CE) gene family.

| **CE FAMILY** | **OIN** | **OSI** | **MAN** | **MAC** | **CMI** | **BBA** | **TOL** | **FGR** | **MGR** | **GCL** | **SSC** | **BCI** | **VER** |
| --- | --- | --- | --- | --- | --- | --- | --- | --- | --- | --- | --- | --- | --- |
| CE1 | 10 | 10 | 16 | 12 | 17 | 17 | 11 | 18 | 28 | 15 | 14 | 17 | 15 |
| CE2 | 0 | 0 | 0 | 0 | 0 | 0 | 0 | 1 | 2 | 0 | 2 | 1 | 1 |
| CE3 | 4 | 3 | 4 | 2 | 2 | 2 | 5 | 9 | 8 | 2 | 2 | 2 | 7 |
| CE4 | 5 | 6 | 4 | 3 | 4 | 3 | 2 | 9 | 12 | 6 | 6 | 5 | 8 |
| CE5 | 1 | 2 | 3 | 2 | 4 | 5 | 2 | 13 | 17 | 1 | 8 | 11 | 15 |
| CE7 | 0 | 0 | 1 | 0 | 0 | 0 | 0 | 0 | 0 | 1 | 0 | 1 | 0 |
| CE8 | 0 | 0 | 0 | 0 | 0 | 0 | 0 | 7 | 1 | 1 | 5 | 5 | 8 |
| CE9 | 2 | 1 | 1 | 1 | 1 | 2 | 1 | 1 | 1 | 2 | 2 | 2 | 1 |
| CE10 | 27 | 24 | 39 | 33 | 33 | 37 | 34 | 65 | 47 | 32 | 45 | 59 | 35 |
| CE12 | 1 | 0 | 0 | 2 | 2 | 1 | 2 | 5 | 3 | 1 | 5 | 5 | 4 |
| CE14 | 1 | 1 | 2 | 2 | 1 | 1 | 1 | 1 | 1 | 1 | 1 | 1 | 0 |
| CE15 | 0 | 0 | 0 | 0 | 0 | 0 | 0 | 0 | 1 | 0 | 1 | 0 | 1 |
| CE16 | 1 | 1 | 2 | 1 | 1 | 2 | 1 | 5 | 2 | 0 | 6 | 7 | 3 |
| **TOTAL** | **52** | **48** | **72** | **58** | **65** | **70** | **59** | **134** | **123** | **62** | **97** | **116** | **94** |

**Supplementary Table S3d.** Distribution of Carbohydrate-degrading enzymes among *Ophiocordyceps indica* and other twelve fungal genomes arranged by Glycosyltransferase (GT) gene family.

| **GT FAMILY** | **OIN** | **OSI** | **MAN** | **MAC** | **CMI** | **BBA** | **TOL** | **FGR** | **MGR** | **GCL** | **SSC** | **BCI** | **VER** |
| --- | --- | --- | --- | --- | --- | --- | --- | --- | --- | --- | --- | --- | --- |
| GT1 | 10 | 5 | 11 | 10 | 8 | 8 | 8 | 14 | 11 | 5 | 8 | 9 | 10 |
| GT2 | 12 | 10 | 10 | 11 | 12 | 13 | 11 | 15 | 11 | 12 | 16 | 13 | 16 |
| GT2_Cellulose_synt | 0 | 0 | 0 | 0 | 0 | 0 | 0 | 0 | 0 | 2 | 0 | 0 | 0 |
| GT3 | 1 | 1 | 1 | 1 | 1 | 1 | 1 | 1 | 1 | 1 | 1 | 1 | 1 |
| GT4 | 7 | 3 | 6 | 6 | 4 | 4 | 3 | 5 | 4 | 6 | 5 | 6 | 6 |
| GT5 | 0 | 0 | 0 | 0 | 0 | 0 | 0 | 0 | 0 | 0 | 0 | 0 | 0 |
| GT8 | 2 | 3 | 5 | 6 | 1 | 2 | 2 | 6 | 2 | 2 | 6 | 7 | 4 |
| GT15 | 5 | 6 | 4 | 4 | 3 | 3 | 4 | 4 | 4 | 3 | 4 | 4 | 4 |
| GT17 | 0 | 1 | 2 | 1 | 4 | 3 | 1 | 1 | 1 | 0 | 2 | 2 | 0 |
| GT20 | 3 | 3 | 4 | 4 | 4 | 4 | 3 | 3 | 3 | 3 | 3 | 3 | 3 |
| GT21 | 1 | 1 | 2 | 2 | 1 | 1 | 1 | 1 | 2 | 2 | 1 | 1 | 1 |
| GT22 | 4 | 3 | 4 | 5 | 4 | 4 | 4 | 4 | 4 | 4 | 4 | 4 | 3 |
| GT23 | 0 | 1 | 0 | 0 | 1 | 0 | 1 | 0 | 0 | 1 | 0 | 0 | 0 |
| GT24 | 1 | 1 | 1 | 1 | 1 | 1 | 1 | 1 | 1 | 1 | 1 | 1 | 1 |
| GT25 | 4 | 3 | 6 | 4 | 2 | 8 | 3 | 0 | 7 | 0 | 3 | 2 | 0 |
| GT27 | 0 | 0 | 0 | 0 | 0 | 0 | 0 | 0 | 0 | 0 | 0 | 0 | 0 |
| GT28 | 1 | 1 | 1 | 1 | 1 | 1 | 1 | 1 | 1 | 1 | 1 | 1 | 0 |
| GT30 | 1 | 0 | 0 | 0 | 0 | 0 | 0 | 0 | 0 | 0 | 0 | 0 | 0 |
| GT31 | 2 | 3 | 5 | 5 | 1 | 3 | 4 | 2 | 2 | 0 | 2 | 2 | 5 |
| GT32 | 11 | 8 | 13 | 9 | 7 | 7 | 10 | 6 | 10 | 4 | 6 | 8 | 3 |
| GT33 | 1 | 1 | 1 | 1 | 1 | 1 | 1 | 1 | 1 | 1 | 1 | 0 | 1 |
| GT34 | 4 | 3 | 5 | 5 | 4 | 5 | 4 | 5 | 2 | 3 | 2 | 1 | 5 |
| GT35 | 1 | 1 | 1 | 1 | 1 | 1 | 1 | 1 | 1 | 1 | 1 | 1 | 1 |
| GT39 | 4 | 3 | 4 | 3 | 3 | 3 | 3 | 3 | 3 | 3 | 3 | 3 | 3 |
| GT41 | 1 | 0 | 1 | 1 | 1 | 1 | 0 | 1 | 1 | 0 | 0 | 0 | 0 |
| GT44 | 0 | 0 | 0 | 0 | 0 | 0 | 0 | 0 | 0 | 0 | 0 | 0 | 0 |
| GT45 | 0 | 0 | 0 | 0 | 0 | 0 | 0 | 0 | 0 | 0 | 0 | 0 | 0 |
| GT48 | 1 | 1 | 1 | 1 | 1 | 1 | 1 | 1 | 1 | 1 | 1 | 1 | 1 |
| GT50 | 1 | 1 | 1 | 1 | 1 | 1 | 1 | 1 | 1 | 1 | 1 | 1 | 1 |
| GT51 | 0 | 0 | 0 | 0 | 0 | 0 | 0 | 0 | 0 | 0 | 0 | 0 | 0 |
| GT54 | 0 | 0 | 1 | 0 | 1 | 0 | 0 | 1 | 0 | 0 | 0 | 0 | 1 |
| GT56 | 0 | 0 | 0 | 0 | 0 | 0 | 0 | 0 | 0 | 0 | 0 | 0 | 0 |
| GT57 | 4 | 3 | 3 | 3 | 3 | 1 | 3 | 3 | 3 | 3 | 3 | 3 | 2 |
| GT58 | 2 | 1 | 1 | 1 | 1 | 1 | 1 | 1 | 1 | 1 | 1 | 1 | 1 |
| GT59 | 1 | 1 | 1 | 0 | 1 | 1 | 1 | 1 | 1 | 1 | 1 | 0 | 1 |
| GT61 | 0 | 0 | 0 | 0 | 0 | 0 | 0 | 0 | 0 | 0 | 0 | 0 | 0 |
| GT62 | 3 | 3 | 3 | 3 | 3 | 3 | 3 | 3 | 3 | 3 | 3 | 3 | 3 |
| GT64 | 0 | 0 | 1 | 1 | 1 | 2 | 3 | 2 | 0 | 0 | 0 | 0 | 1 |
| GT65 | 0 | 0 | 0 | 0 | 0 | 0 | 0 | 0 | 0 | 0 | 0 | 0 | 0 |
| GT66 | 1 | 1 | 1 | 1 | 1 | 1 | 1 | 1 | 1 | 1 | 1 | 1 | 1 |
| GT68 | 0 | 0 | 0 | 0 | 0 | 0 | 0 | 0 | 0 | 0 | 2 | 3 | 0 |
| GT69 | 4 | 3 | 2 | 3 | 3 | 3 | 2 | 2 | 4 | 4 | 2 | 3 | 2 |
| GT71 | 2 | 2 | 1 | 0 | 1 | 1 | 0 | 4 | 4 | 4 | 2 | 5 | 0 |
| GT76 | 1 | 1 | 1 | 1 | 1 | 1 | 1 | 1 | 1 | 1 | 1 | 1 | 1 |
| GT77 | 0 | 0 | 1 | 1 | 0 | 0 | 0 | 1 | 0 | 0 | 0 | 0 | 0 |
| GT81 | 0 | 0 | 0 | 0 | 0 | 0 | 0 | 0 | 0 | 0 | 0 | 0 | 0 |
| GT83 | 1 | 0 | 0 | 0 | 0 | 0 | 0 | 0 | 0 | 0 | 0 | 0 | 0 |
| GT90 | 6 | 6 | 6 | 6 | 7 | 6 | 6 | 6 | 7 | 5 | 5 | 5 | 5 |
| GT92 | 0 | 0 | 0 | 0 | 0 | 0 | 0 | 0 | 0 | 0 | 1 | 1 | 0 |
| **TOTAL** | **103** | **84** | **111** | **103** | **90** | **96** | **90** | **103** | **99** | **80** | **94** | **97** | **87** |

**Supplementary Table S3e.** Distribution of Carbohydrate-degrading enzymes among *Ophiocordyceps indica* and other twelve fungal genomes arranged by Polysaccharide Lyase (PL) gene family.

| **PL**  **FAMILY** | **OIN** | **OSI** | **MAN** | **MAC** | **CMI** | **BBA** | **TOL** | **FGR** | **MGR** | **GCL** | **SSC** | **BCI** | **VER** |
| --- | --- | --- | --- | --- | --- | --- | --- | --- | --- | --- | --- | --- | --- |
| PL1 | 0 | 0 | 0 | 0 | 0 | 0 | 0 | 0 | 0 | 0 | 0 | 0 | 2 |
| PL1_1 | 0 | 0 | 0 | 0 | 0 | 0 | 0 | 0 | 0 | 0 | 0 | 0 | 0 |
| PL1_10 | 0 | 0 | 0 | 0 | 0 | 0 | 0 | 1 | 1 | 0 | 0 | 0 | 1 |
| PL1_11 | 0 | 0 | 0 | 0 | 0 | 0 | 0 | 0 | 0 | 0 | 0 | 0 | 0 |
| PL1_12 | 0 | 0 | 0 | 0 | 0 | 0 | 0 | 0 | 0 | 0 | 0 | 0 | 0 |
| PL1_2 | 0 | 0 | 0 | 0 | 0 | 0 | 0 | 0 | 0 | 0 | 0 | 0 | 2 |
| PL1_3 | 0 | 0 | 0 | 0 | 0 | 0 | 0 | 0 | 0 | 0 | 0 | 0 | 0 |
| PL1_4 | 0 | 0 | 0 | 0 | 0 | 0 | 0 | 4 | 0 | 1 | 3 | 4 | 5 |
| PL1_5 | 0 | 0 | 0 | 0 | 0 | 0 | 0 | 0 | 0 | 0 | 0 | 0 | 0 |
| PL1_6 | 0 | 0 | 0 | 0 | 0 | 0 | 0 | 0 | 0 | 0 | 0 | 0 | 0 |
| PL1_7 | 0 | 0 | 0 | 0 | 0 | 0 | 0 | 3 | 1 | 1 | 1 | 2 | 4 |
| PL1_8 | 0 | 0 | 0 | 0 | 0 | 0 | 0 | 0 | 0 | 0 | 0 | 0 | 0 |
| PL1_9 | 0 | 0 | 0 | 0 | 0 | 0 | 0 | 1 | 0 | 0 | 0 | 0 | 2 |
| PL3 | 0 | 0 | 0 | 0 | 0 | 0 | 0 | 0 | 0 | 0 | 0 | 0 | 0 |
| PL3_1 | 0 | 0 | 0 | 0 | 0 | 0 | 0 | 0 | 0 | 0 | 0 | 0 | 0 |
| PL3_2 | 0 | 0 | 0 | 0 | 0 | 0 | 0 | 7 | 1 | 1 | 0 | 2 | 12 |
| PL3_3 | 0 | 0 | 0 | 0 | 0 | 0 | 0 | 0 | 0 | 0 | 0 | 0 | 0 |
| PL3_4 | 0 | 0 | 0 | 0 | 0 | 0 | 0 | 0 | 0 | 0 | 0 | 0 | 0 |
| PL3_5 | 0 | 0 | 0 | 0 | 0 | 0 | 0 | 0 | 0 | 0 | 0 | 0 | 0 |
| PL4 | 0 | 0 | 0 | 0 | 0 | 0 | 0 | 0 | 0 | 0 | 0 | 0 | 0 |
| PL4_1 | 0 | 0 | 0 | 0 | 0 | 0 | 0 | 1 | 0 | 0 | 0 | 0 | 1 |
| PL4_2 | 0 | 0 | 0 | 0 | 0 | 0 | 0 | 0 | 0 | 0 | 0 | 0 | 0 |
| PL4_3 | 0 | 0 | 0 | 0 | 0 | 0 | 0 | 2 | 1 | 0 | 0 | 0 | 2 |
| PL4_4 | 0 | 0 | 0 | 0 | 0 | 0 | 0 | 0 | 0 | 0 | 0 | 0 | 0 |
| PL4_5 | 0 | 0 | 0 | 0 | 0 | 0 | 0 | 0 | 0 | 1 | 0 | 0 | 2 |
| PL6 | 0 | 0 | 0 | 0 | 0 | 0 | 0 | 0 | 0 | 0 | 0 | 0 | 0 |
| PL6_1 | 0 | 0 | 0 | 0 | 0 | 0 | 0 | 0 | 0 | 0 | 0 | 0 | 0 |
| PL6_2 | 0 | 0 | 0 | 0 | 0 | 0 | 0 | 0 | 0 | 0 | 0 | 0 | 0 |
| PL7 | 0 | 0 | 0 | 0 | 0 | 0 | 0 | 0 | 0 | 0 | 0 | 0 | 0 |
| PL7_1 | 0 | 0 | 0 | 0 | 0 | 0 | 0 | 0 | 0 | 0 | 0 | 0 | 0 |
| PL7_2 | 0 | 0 | 0 | 0 | 0 | 0 | 0 | 0 | 0 | 0 | 0 | 0 | 0 |
| PL7_3 | 0 | 0 | 0 | 0 | 0 | 0 | 0 | 0 | 0 | 0 | 0 | 0 | 0 |
| PL7_4 | 0 | 2 | 1 | 1 | 1 | 1 | 1 | 0 | 0 | 0 | 1 | 1 | 0 |
| PL7_5 | 0 | 0 | 0 | 0 | 0 | 0 | 0 | 0 | 0 | 0 | 0 | 0 | 0 |
| PL8 | 0 | 0 | 1 | 1 | 1 | 1 | 1 | 0 | 0 | 0 | 0 | 0 | 0 |
| PL8_1 | 0 | 0 | 0 | 0 | 0 | 0 | 0 | 0 | 0 | 0 | 0 | 0 | 0 |
| PL8_2 | 0 | 0 | 0 | 0 | 0 | 0 | 0 | 0 | 0 | 0 | 0 | 0 | 0 |
| PL8_3 | 0 | 0 | 0 | 0 | 0 | 0 | 0 | 0 | 0 | 0 | 0 | 0 | 0 |
| PL9 | 0 | 0 | 0 | 0 | 0 | 0 | 0 | 0 | 0 | 0 | 0 | 0 | 0 |
| PL9_1 | 0 | 0 | 0 | 0 | 0 | 0 | 0 | 0 | 0 | 0 | 0 | 0 | 1 |
| PL9_2 | 0 | 0 | 0 | 0 | 0 | 0 | 0 | 0 | 0 | 0 | 0 | 0 | 0 |
| PL9_3 | 0 | 0 | 0 | 0 | 0 | 0 | 0 | 1 | 0 | 0 | 0 | 0 | 2 |
| PL10 | 0 | 0 | 0 | 0 | 0 | 0 | 0 | 0 | 0 | 0 | 0 | 0 | 0 |
| PL11 | 0 | 0 | 0 | 0 | 0 | 0 | 0 | 0 | 0 | 0 | 0 | 0 | 1 |
| PL11_1 | 0 | 0 | 0 | 0 | 0 | 0 | 0 | 0 | 0 | 0 | 0 | 0 | 0 |
| PL11_2 | 0 | 0 | 0 | 0 | 0 | 0 | 0 | 0 | 0 | 0 | 0 | 0 | 0 |
| PL12 | 0 | 0 | 0 | 0 | 1 | 0 | 0 | 0 | 0 | 0 | 0 | 0 | 0 |
| PL15 | 0 | 0 | 0 | 0 | 0 | 0 | 0 | 0 | 0 | 0 | 0 | 0 | 0 |
| PL18 | 0 | 0 | 0 | 0 | 0 | 0 | 0 | 0 | 0 | 0 | 0 | 0 | 0 |
| PL20 | 0 | 0 | 3 | 2 | 1 | 0 | 0 | 1 | 1 | 0 | 0 | 0 | 0 |
| PL22 | 0 | 0 | 0 | 0 | 0 | 0 | 0 | 0 | 0 | 0 | 0 | 1 | 0 |
| PL22_1 | 0 | 0 | 0 | 0 | 0 | 0 | 0 | 0 | 0 | 0 | 0 | 0 | 0 |
| PL22_2 | 0 | 0 | 0 | 0 | 0 | 0 | 0 | 0 | 0 | 0 | 0 | 0 | 0 |
| PL24 | 0 | 0 | 0 | 0 | 0 | 0 | 0 | 0 | 0 | 0 | 0 | 0 | 0 |
| PL26 | 0 | 0 | 0 | 0 | 0 | 0 | 0 | 1 | 1 | 2 | 0 | 0 | 1 |
| PL27 | 0 | 0 | 0 | 0 | 0 | 0 | 0 | 0 | 1 | 0 | 0 | 0 | 0 |
| **TOTAL** | **0** | **2** | **5** | **4** | **4** | **2** | **2** | **22** | **7** | **5** | **5** | **10** | **38** |

**Supplementary Table 4** Grading of commercial *Ophiocordyceps sinensis.*

| **Grade** | **No. of Pieces** | **Description** |
| --- | --- | --- |
| A++ | G-2000 | 2000 pcs/kg |
| A+ | G-3000 | 3000 pcs/kg |
| A | G-4000 | 4000 pcs/kg |
| B+ | G-5000 | 5000 pcs/kg |
| B | G-6000 | 6000 pcs/kg |
| C | G-7000 | 7000 pcs/kg |
| D | G-R | Rejects & Broken Pcs |

Source: http://dragonherbsbhutan.com/cordyceps-sinensis/grading-of-cordyceps/
